# Prognostic and Clinicopathological Significance of Telomerase Reverse Transcriptase Upregulation in Oral Cancer: A Systematic Review and Meta-Analysis

**DOI:** 10.3390/cancers14153673

**Published:** 2022-07-28

**Authors:** Miguel Ángel González-Moles, Eloísa Moya-González, Alberto García-Ferrera, Paola Nieto-Casado, Pablo Ramos-García

**Affiliations:** 1 School of Dentistry, University of Granada, 18011 Granada, Spain; eloisamg@correo.ugr.es (E.M.-G.); albertogf6@correo.ugr.es (A.G.-F.); paolanieto@correo.ugr.es (P.N.-C.); 2 Instituto de Investigación Biosanitaria ibs.GRANADA, 18012 Granada, Spain

**Keywords:** telomerase reverse transcriptase (TERT), hTERT, hEST2, replicative immortality, oral cancer, prognosis, biomarker, systematic review, meta-analysis

## Abstract

**Simple Summary:**

Hanahan and Weinberg proposed to the scientific community a series of distinctive characteristics that all malignant neoplastic cells should possess, which had an enormous impact on the scientific community. One of the most relevant hallmarks of cancer involves the ability of tumor cells to acquire replicative immortality, achieved essentially through the maintenance of telomere length, dependent essentially on telomerase activity. Telomerase lengthens telomeres through the reverse transcription of a 6-bp telomere repeat sequence at the 3′ end of the telomere, which requires the enzymatic component of telomerase, i.e., the telomerase reverse transcriptase (TERT). This mechanism is essential for tumor cells to survive for a long time, and, as has been pointed out, it is frequently activated in malignant cells. This is the first systematic review and meta-analysis, based on 21 studies and 1698 oral squamous cell carcinoma (OSCC) patients, demonstrating that TERT protein overexpression behaves as a prognostic biomarker, significantly associated with poor survival in oral cancer.

**Abstract:**

The aim of this systematic review and meta-analysis was to evaluate the current evidence on the prognostic and clinicopathological significance value of telomerase reverse transcriptase (TERT) upregulation in patients with oral squamous cell carcinoma (OSCC). PubMed, Embase, Web of Science, and Scopus were searched for studies published before April 2022, not restricted by date or publication language. The methodological quality of primary-level studies was critically assessed using the Quality in Prognosis Studies (QUIPS) tool. We carried out meta-analyses, explored heterogeneity and its sources, and performed subgroup, meta-regression, sensitivity, and small-study effects analyses. Twenty-one studies (1698 patients) met inclusion criteria. TERT protein overexpression was significantly associated with worse overall survival (hazard ratio [HR] = 3.01, 95% CI = 1.70–5.35, *p* < 0.001), disease-free survival (HR = 4.03, 95% CI = 1.80–9.05, *p* = 0.001), and higher histological grade OSCC (odds ratio [OR] = 3.20, 95% CI = 1.83–5.62, *p* < 0.001). These large effect sizes were consistently obtained by homogeneous subgroups (*p* > 0.10, I^2^ = 0.0, respectively), which reflects a high quality of evidence. On the other hand, TERT gene mutations obtained constantly nonsignificant null effect sizes for all outcomes investigated, evidencing no prognostic or clinicopathological value. In conclusion, our findings indicate that TERT upregulation is a prognostic indicator of poor survival in oral cancer. Our findings support the immunohistochemical assessment of TERT overexpression, which could probably be incorporated into the prognostic evaluation of OSCC.

## 1. Introduction

In 2000, Hanahan and Weinberg [1] proposed to the scientific community a series of distinctive characteristics that all neoplastic cells should possess, regardless of the origin of the tumor tissue. This proposal was complemented in 2011 [2] with new characteristics that jointly have received the denomination of hallmarks of cancer. The final proposal of these authors includes a series of hallmarks (sustaining proliferative signaling, resistance to cell death, evading growth suppressors, angiogenesis, invasion and metastasis, deregulating cellular energetics, destruction of immune evasion and replicative immortality, and avoiding immune destruction), and two enabling features (genome instability and mutation, and tumor-promoting inflammation). The papers of Hanahan and Weinberg have had an enormous impact on the scientific community and indisputably marked lines of research in different types of tumors that have sought to evaluate the extent to which each of these hallmarks is of special value in terms of diagnosis, prognosis, or treatment. However, it should be emphasized that to date there is very little evidence on the importance of these hallmarks in oral cancer. One of the most relevant hallmarks of cancer involves the ability of tumor cells to acquire replicative immortality, which they achieve essentially through the maintenance of telomere length, dependent essentially on telomerase activity. Telomerase is a ribonucleoprotein complex enzymatically active in 80–90% of all malignancies [3,4,5]. In the absence of telomerase activity, telomeres are gradually shortened after each cell-replication cycle [6], which results in the loss of protection that they exert over the rest of the chromosome, generating alterations in chromosome morphology: disruptions, aberrant fusions, chromosomes with more than one centromere, activation of oncogenes, and alteration of tumor suppressor genes, with a subsequent increase in oncogenic signals that in turn activate a DNA damage response, inducing senescence [7]. Telomerase activity during malignant transformation prevents telomere shortening and promotes cellular immortality, as we have discussed, a hallmark of cancer [2,8,9]. Telomerase lengthens telomeres through the reverse transcription of a 6-bp telomere repeat sequence at the 3′ end of the telomere, which requires the enzymatic component of telomerase, known as telomerase reverse transcriptase (TERT) [10,11,12,13]. This mechanism is essential for tumor cells to survive for a long time, and, as has been pointed out, it is frequently activated in malignant cells. Although in recent years a considerable number of primary-level studies have been published on the importance of the telomeric length preservation mechanism in oral cancer and its relevance in the acquisition of replicative immortality, there is no study with an evidence-based design (i.e., systematic review and meta-analysis) on the frequency of this phenomenon and its prognostic implications in oral cancer. This knowledge would be relevant, since in recent years telomerase is being considered as a potential therapeutic target in cancer [3].

Consequently, and based on the previous comments, we carried out a systematic review and meta-analysis on the clinical and prognostic significance of the replicative immortality linked to telomerase activation, essentially by activation of one of its most relevant components—TERT—in oral cancer.

## 2. Materials and Methods

This systematic review and meta-analysis closely complied with PRISMA and MOOSE reporting guidelines, closely followed the criteria of the Cochrane Prognosis Methods Group [14] and Cochrane Collaboration criteria [15], and was conducted and validated in accordance with AMSTAR2 guidelines [16].

### 2.1. Protocol

A study protocol was first designed and registered in the PROSPERO International Prospective Register of Systematic Reviews (www.crd.york.ac.uk/PROSPERO; ID338697; CRD42022338697; accessed on 31 May 2022), in order to minimize the risk of bias and reinforcing the transparency, precision, and integrity of our study. The protocol complied with PRISMA-P reporting guidelines [17].

### 2.2. Search Strategy

We searched Medline (through PubMed), Embase, Web of Science, and Scopus databases for studies published before April 2022 (upper limit), with no earlier date limit. Searches were conducted by combining the thesaurus terms used by the databases (i.e., MeSH and Emtree) with free terms (Table S1, Supplementary Materials), designed and built to maximize sensitivity. An additional screening was performed hand-searching the reference lists of retrieved included studies. All references were managed using Mendeley v.1.19.8 (Elsevier, Amsterdam, The Netherlands). The duplicate-removal process was also driven using this software.

### 2.3. Eligibility Criteria

Inclusion criteria were: original primary-level studies, without restrictions by language, publication date, study design, follow-up periods, geographical area, age, or sex; evaluation of TERT upregulation (through TERT gene mutations, mRNA or protein overexpression) in samples from OSCC; and analysis of the association with at least one of the following prognostic and/or clinicopathological outcomes: overall survival (OS), disease-free survival (DFS), tumor size, N status, clinical stage, or histological grade. OS was defined as the time elapsed from date of diagnosis/surgery to date of death by any cause. DFS was defined as the time elapsed from diagnosis/surgery to the detection of locoregional or distant recurrence or to death without recurrence. Given the lack of international consensus standards to define survival end points in oncology research, any study using the terms OS and DFS was included, or by using other terms in compliance with our precedent definitions.

Exclusion criteria were: retracted articles, preclinical research (in vitro research or in vivo animal experimentation), case reports, editorials, letters, meeting abstracts, personal opinions, comments, or book chapters, or secondary/tertiary-evidence level studies (systematic reviews, meta-analyses, scoping reviews, umbrella or overviews of reviews, etc.); squamous cell carcinomas from anatomic areas distinct to the oral cavity, and/or tumors of different histopathological lineage; no analysis of the main prognostic or clinicopathological outcomes of interest; lacking or insufficient data for the estimation of statistical effect-size metrics with their corresponding confidence intervals; and overlapping interstudy populations, determined by verifying the authors’ names and affiliations, source of patients, and recruitment periods. When potential overlapping populations were identified, the reports providing more complete datasets were included. 

### 2.4. Study-Selection Process

Eligibility criteria were independently applied by a team of three blinded authors (EMG, AGF, and PNC). Any discrepancies were resolved by consensus with a fourth author (PRG). The records were selected across two phases: phase I involved screening titles and abstracts and phase II full-text reading of the selected articles, excluding those that did not meet the eligibility criteria. Evaluators were first jointly trained and calibrated for the process of identification and selection of studies, performing an initial screening round (50 papers each). An optimal interagreement proportional score (relative frequency of agreement = 99.91%) was finally obtained. Interrater reliability was also measured using Cohen’s kappa statistic, obtaining almost perfect agreement (κ = 0.95).

### 2.5. Data Extraction

Authors independently extracted data from the selected articles, filling a data collection form in a standardized manner using Excel (v.16/2018, Microsoft, Redmond, WA, USA). Datasets extracted were secondarily jointly cross-checked, solving discrepancies by consensus. Data expressed as order statistics (i.e., median, interquartile range, and/or maximum and minimum values) were computed and transformed, if possible, into means ± standard deviation (SD) using the methods proposed by Luo et al. (2018) and Wan et al. (2014) [18,19]. If it were desirable to combine two or more different datasets expressed as means ± SD from subgroups into a single group, the Cochrane handbook formula was applied [15]. Data were gathered on the first author, language, publication date, country, sample size, cancer subsite, sex and age of patients, tobacco, areca nut, and alcohol consumption, recruitment and follow-up period, study design, experimental methods, and relative frequency of TERT upregulation. Finally, the data required to analyze the outcomes were also recorded for survival (OS and DFS) and clinicopathological variables (T status [T3/T4 vs. T1/T2], N status [N+ vs. N−], clinical stage [III/IV vs. I/II], and histological grade [II/III vs. I]). Furthermore, clinicopathological variables rarely reported in primary-level studies were also ad hoc screened and categorized. We identified and extracted data on the relationships between TERT upregulation and extracapsular spread (extracapsular vs. intracapsular), tumor margins, and perineural and lymphatic invasion (positive vs. negative, respectively). 

### 2.6. Evaluation of the Methodological Quality and Risk of Bias across Primary-Level Studies

The authors critically appraised the methodological quality and risk of bias across primary-level studies using the Quality in Prognosis Studies (QUIPS) tool (developed by members of the Cochrane Prognosis Methods Group [20]). The following six potential bias domains were explored: (1) study participation; (2) study attrition; (3) prognostic factor measurement; (4) outcome measurement; (5) study confounding; and (6) statistical analysis/reporting. The risk of bias was considered low, moderate, or high for each domain. Finally, an overall score was also estimated based on a method previously described by our research group [21,22,23], in order to obtain an overall risk-of-bias score.

### 2.7. Statistical Analysis

TERT upregulation was analyzed as a dichotomous categorical variable according to the scoring systems adopted by primary-level studies. Hazard ratios (HRs) and 95% confidence intervals (CIs) were used for the meta-analysis of prognostic variables due to their time-to-event nature [24]. When authors reported effect-size metrics in their survival analyses, these were directly extracted from the primary-level studies. If HRs and/or 95% CIs were not explicitly provided by the authors, we calculated them using the methods described by Parmar et al. [25] and Tierney et al. [24] When a study gave only survival curves, we extracted the data from Kaplan–Meier curves with Engauge Digitizer 4.1 software (open-source digitizing software developed by M. Mitchell). When HRs were determined in both univariable and multivariable models, data were extracted from the multivariable model, which reflects a greater adjustment for potentially confounding factors. Odds ratios (OR) with their corresponding 95% CIs were estimated and used as an effect-size measure for the meta-analyses of the clinicopathological variables. If authors reported their results as continuous variables with means ± standard deviations, Cohen’s *d* standardized mean differences/SMDs were first calculated and subsequently reexpressed as odds ratios using pertinent conversion methods, i.e.,

(1) $$Log\left(OR\right)=d\frac{\pi }{\sqrt{3}}$$

(based on an assumption that the underlying continuous measurements followed a logistic distribution) [15,26].

All meta-analyses were conducted using the inverse-variance method under a random-effects model (based on the DerSimonian and Laird method). This approach was a priori planned in our study protocol, in order to account for the possibility that there are different underlying effects among study subpopulations (e.g., differences among experimental methods, TERT alterations, or geographical areas). Forest plots were constructed in all meta-analyses performed, in order to graphically represent the effect sizes and for subsequent visual inspection analysis. Heterogeneity between studies was assessed using the χ^2^-based Cochran’s Q test. Given the low statistical power of the Q test, *p* < 0.10 was considered significant. We also applied the Higgins I^2^ statistic to estimate what proportion of the variance in observed effects reflected variation in true effects, rather than sampling error. The percentage of interstudy heterogeneity was quantified, considering values of 50–75% as a moderate-to-high degree of inconsistency [27,28]. Preplanned subgroup meta-analyses by TERT upregulation alterations (i.e., at protein level, mRNA, or gene mutations) and geographical area were performed to identify potential sources of heterogeneity. Furthermore, additional univariable random-effect meta-regression analyses were conducted using the restricted maximum likelihood (REML) method to explore the potential effect of additional study covariates (i.e., follow-up period, age, sex, and tobacco and alcohol use) [29]. Considering the low number of studies with data available for meta-regression analyses, the *p*-values were recalculated using a permutation test based on Monte Carlo simulations [30]. To obtain sufficient precision, the number of permutations was 10,000 [31]. Weighted bubble plots were also constructed to graphically represent the fitted meta-regression lines. Furthermore, two additional statistical analyses were carried out to test the stability and reliability of our meta-analytical results: first, sensitivity analyses were carried out to explore the influence of each primary-level study on the pooled overall estimates [32], repeating sequentially the meta-analyses, omitting one study at a time (“leave-one-out” method); second, small-study effect analyses were carried out to identify potential biases, such as publication bias, constructing funnel plots and using the Egger regression test (performing a linear regression of the effect estimates on their standard errors, weighting by 1/[variance of the effect estimate], considering a *p*_Egger_-value < 0.10 as significant) [33]. 

Finally, the meta-analysis of secondary clinicopathological parameters (i.e., extracapsular spread, tumor margins, perineural and lymphatic invasion) could not be performed due to the low number of observations extracted, jointly with a considerable degree of clinical and methodological heterogeneity. However, due to their potential prognostic implications, an albatross plot was constructed to graphically represent them [34], allowing an approximate examination of their underlying magnitudes of effect. Stata software was used for all statistical analyses (v.16.1, Stata Corp, College Station, TX, USA).

### 2.8. Validation of Methodological Quality

The methodology followed in this systematic review and meta-analysis was designed, critically appraised, and validated using the AMSTAR2 checklist [16], created as an instrument to develop, evaluate, and validate high-quality systematic reviews and meta-analyses through 16 items [16]. The overall confidence on the methodology of a systematic review is rated as “high”, “moderate”, “low”, and “critically low”.

## 3. Results

### 3.1. Results of the Literature Search

**Figure 1:**
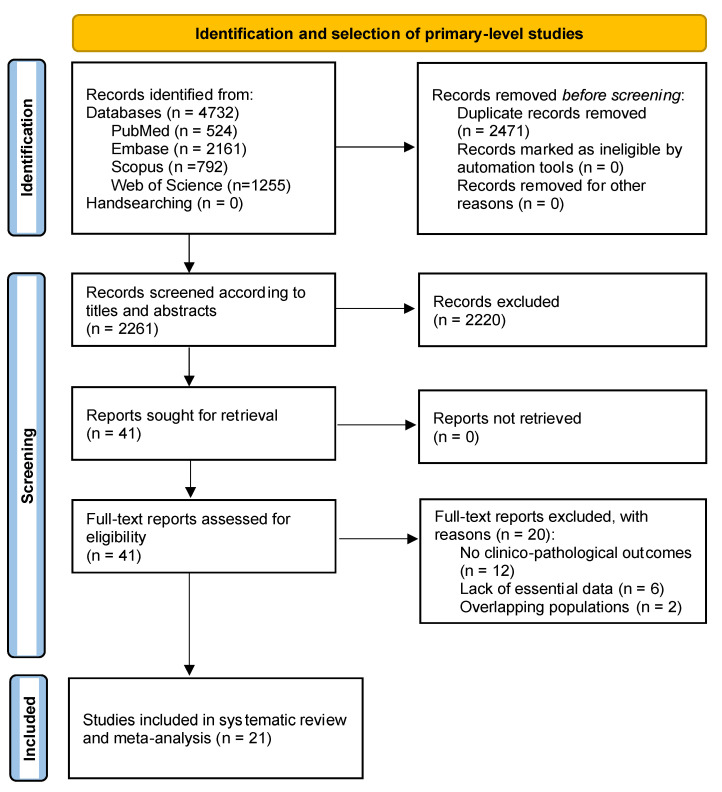
Flow diagram showing the identification and selection process of the study sample, analyzing primary-level studies researching the prognostic and clinicopathological significance TERT upregulation in OSCC.

The flow diagram in Figure 1 depicts the process of identification, screening, and selection of primary-level studies. A total of 4732 records were retrieved: 2161 from Embase, 1255 from Web of Science, 792 from Scopus, and 524 from PubMed. After removal of duplicates, 2261 records were screened according to titles and abstracts, leaving a sample of 41 papers for full-text evaluation (the studies excluded and their exclusion criteria are listed in the Supplementary Materials). Finally, 21 studies meeting all eligibility criteria were included for qualitative evaluation and meta-analysis [35,36,37,38,39,40,41,42,43,44,45,46,47,48,49,50,51,52,53,54,55].

### 3.2. Study Characteristics

Table 1 summarizes the main characteristics of our study sample, and Table S2 (in Supplementary Materials) exhibits in detail the variables gathered from primary-level studies. These 21 studies, recruiting a total of 1698 OSCC patients (range: 30–218 patients), were published between 1999 and 2022 (11/21 [52.38%] published in the last 5 years, i.e., 2017–2022). TERT upregulation was analyzed through gene mutations by 7 studies, at mRNA level (n= 4), and at protein level (n = 10). All studies were observational retrospective cohorts (n = 21) and well distributed across worldwide geographic regions (Asia [n = 8], Europe [n = 8], North America [n = 3], and South America [n = 1]).

### 3.3. Qualitative Evaluation

**Figure 2:**
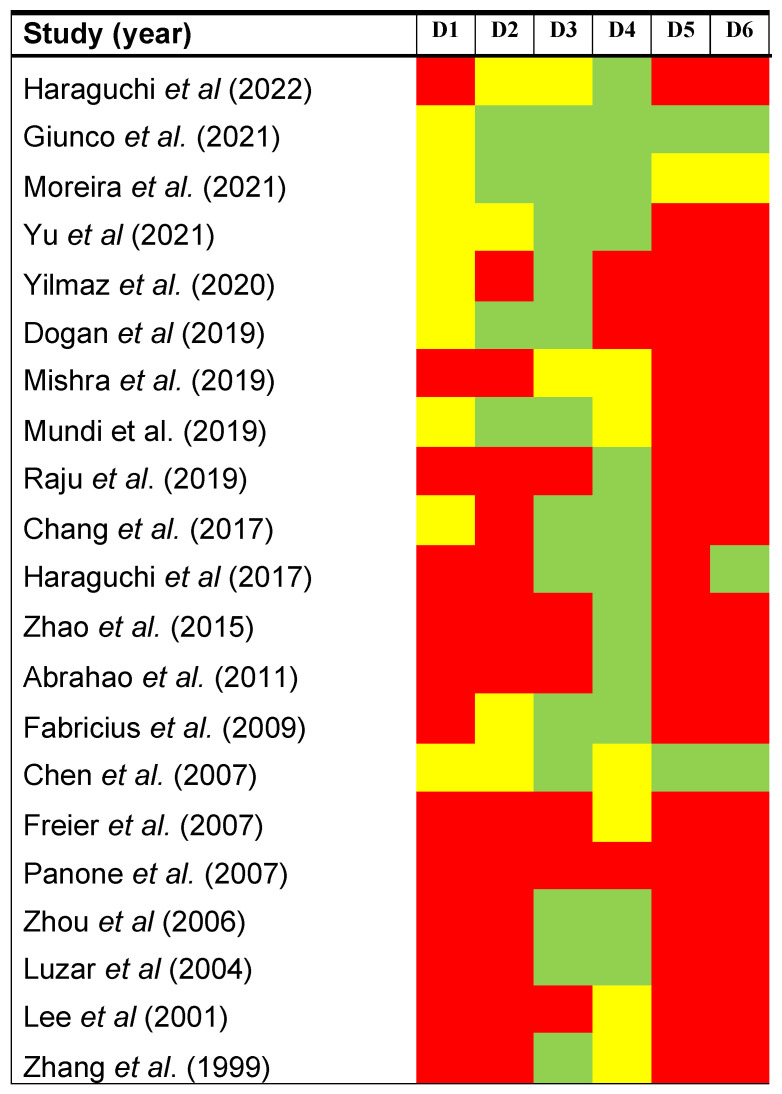
Quality plot depicting the risk of potential bias across primary-level studies, assessed using the Quality in Prognosis Studies tool (QUIPS) developed by the Cochrane Prognosis Methods Group, which considers the following domains: (D1) study participation, (d2) study attrition, (d3) prognostic factor measurement, (d4) outcome measurement, (d5) study confounding, and (d6) statistical analysis and reporting. Risk of bias was classified as low (green), moderate (yellow), or high (red) for each domain.

The following results were derived from the evaluation of the methodological quality and risk of bias across primary-level studies using the QUIPS tool (Figure 2), which considers potential sources of bias in six domains:

*Study participation.* The risk of this bias was high in 61.90% of the reviewed studies and moderate in 38.10%. Studies reporting an inadequate description of their samples (age/sex distribution, oral cancer subsites, etc.) or clinical setting (place and period of recruitment) were considered potentially biased.

*Study attrition.* The risk of this bias was high in 61.90% of the studies, moderate in 19.05%, and low in 19.05%. Some studies did not report essential information on the follow-up period (i.e., mean ± SD, median, IQR, and/or range) and none reported any attempt to collect information and reasons for patients lost to follow-up, or the description of their characteristics, necessary to rule out potential risk of bias in this domain.

*Prognostic factor measurement.* The risk of this bias was high in 28.57% of the studies, moderate in 9.53%, and low in 61.90%. A high risk of potential bias was associated with insufficient information on scoring systems, cutoff points or missing data related to essential aspects of the experimental methods (e.g., anti-TERT antibody). Some immunohistochemical studies reported a labeling index score, the product of an adjustment between intensity and cell count. Although in our opinion this method lacks translational potential, these studies were not critically downgraded.

*Outcome measurement.* The risk of this bias was high in 14.29% of the studies, moderate in 28.57%, and low in 57.14%. The most frequent potential biases were the non-definition of survival parameters in spite of the lack of international consensus on survival end points in cancer research and insufficient reporting on the clinicopathological system adopted (e.g., the edition of the AJCC/UICC TNM staging system, subject to periodic changes). 

*Study confounding.* The risk of this bias was high in 85.71% of the studies, moderate in 4.76%, and low in 9.53%. The most frequent potential biases were the failure to consider potential confounders factors in the study design, to measure all potential confounders (e.g., tobacco or alcohol consumption), or to report adjusted data through multivariable statistical regression analyses.

*Statistical analysis and reporting.* The risk of this bias was high in 80.95% of the studies, moderate in 4.76%, and low in 14.29%. The most frequent biases were statistical models not based on effect-size metrics (e.g., HR or OR) with 95% CIs. It should be remarked that the simple reporting of *p*-values is much less informative on magnitude, precision, and direction of an effect. The most serious potential biases detected were incorrect statistical analyses and errors in the study reporting, offering misleading results and conclusions. 

### 3.4. Quantitative Evaluation (Meta-Analysis)

#### 3.4.1. Association between TERT Upregulation and Prognostic Variables

**Figure 3:**
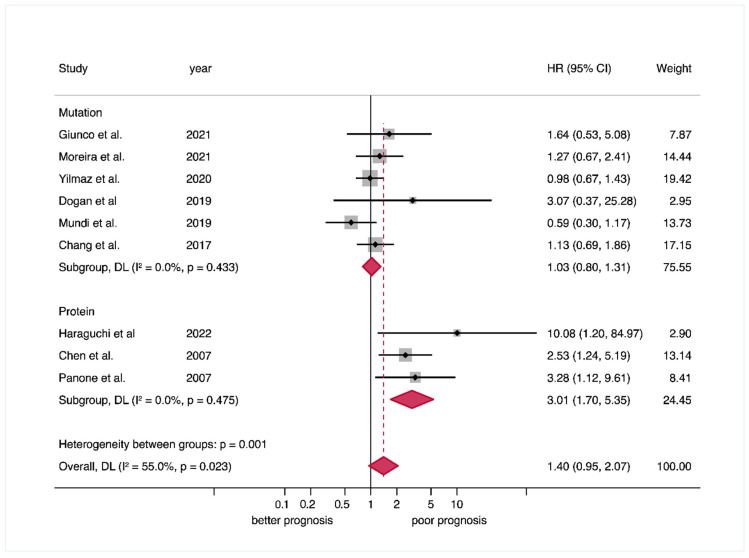
Forest plot graphically representing the meta-analysis on the association between TERT upregulation (stratified by alterations, i.e., gene mutations vs. protein overexpression) and OS in patients with OSCC. Random-effects model, inverse-variance weighting (based on the DerSimonian and Laird method). An HR > 1 suggests that TERT upregulation is associated with poor prognosis. Diamonds indicate pooled HRs with their corresponding 95% CIs. Abbreviations: TERT, telomerase reverse transcriptase; OS, overall survival; OSCC, oral squamous cell carcinoma; HR, hazard ratio; CI, confidence interval.

*Overall survival (OS).* Close to significant results were found between TERT upregulation and poor OS (HR = 1.40, 95% CI = 0.95-2.07, *p* = 0.001), and moderate heterogeneity was present (*p* = 0.02, I^2^ = 55.0%). After the stratified meta-analysis by type of alteration, i.e., mutations vs. protein overexpression, the subgroups were notably homogeneous and separately more comparable (*p* = 0.48/I^2^ = 0.0%, and *p* = 0.43/I^2^ = 0.0%, respectively). Protein overexpression was significantly associated with poor mortality, showing a large effect size (HR = 3.01, 95% CI = 1.70–5.35, *p* < 0.001), also significantly higher than gene mutations (*p* = 0.001), which showed a null effect size (HR = 1.03, 95% CI = 0.80–1.31, *p* = 0.83) (Table 2, Figure 3). Finally, the stratified meta-analysis by geographical area did not find significant differences and showed considerable heterogeneity for the Asian subgroup (*p* = 0.04/I^2^ = 69.4%) (Table 2, Figure S1, Supplementary Materials). These relevant meta-analytical findings indicate that TERT alterations do not all have the same prognostic value, protein overexpression showing the best prognostic performance, representing the most relevant explanatory source of heterogeneity in this meta-analysis.

*Disease-free survival (DFS).* Similar results were found for this parameter, where TERT overexpression was significantly associated with poor DFS (HR = 4.03, 95% CI = 1.80–9.05, *p* = 0.001), while gene mutations showed a nonsignificant and reduced effect size (HR = 1.13, 95% CI = 0.81–1.59, *p* = 0.46). Differences in mRNA expression levels also showed a significant, though imprecise, prognostic value, being assessed only by a single primary-level study (HR = 3.79, 95% CI = 1.03–13.98, *p* = 0.05) (Table 2, Figure S7, Supplementary Materials). 

#### 3.4.2. Association between TERT Upregulation and Clinicopathological Variables

TERT upregulation and/or stratified meta-analyses by alteration (gene mutation vs. protein overexpression) were not significantly associated with T status (OR = 1.15, 95% CI = 0.66–2.03, *p* = 0.62), N status (OR = 1.25, 95% CI = 0.62–2.50, 0.54), or clinical stage (OR = 1.33, 95% CI = 0.41–4.34, *p* = 0.64) (Table 2, Figures S8–S10, Supplementary Materials). Finally, a significant association was found between TERT overexpression and higher histological grade, showing a large effect size (OR = 3.20, 95% CI = 1.83–5.62, *p* < 0.001) (Table 2, Figure S11, Supplementary Materials).

#### 3.4.3. Association between TERT Upregulation and Clinicopathological Variables Not Included in Meta-Analysis

Meta-analysis was not performed for the association between TERT upregulation and secondary clinicopathological variables (extracapsular spread, tumor margins, perineural and lymphatic invasion), where a very low number of primary-level studies reported heterogeneous datasets. However, all were included in an albatross plot (Figure S30, Supplementary Materials) and considered separately in the narrative synthesis. These variables were investigated only for TERT gene mutations, and all observations presented nonsignificant results, which is consistent with our meta-analytical findings.

### 3.5. Quantitative Evaluation (Secondary Analyses)

#### 3.5.1. Meta-Regression Analysis

The potential impact of additional study covariates—follow-up period, sex, age, tobacco and alcohol consumption—on the association between TERT upregulation and OS was analyzed, and no significant differences were found (*p* > 0.05, respectively) (Table 2, Figures S2–S6, Supplementary Materials).

#### 3.5.2. Sensitivity Analysis

The results were stable for prognostic variables and for histological grade parameter, where no substantial changes were observed after the sequential repetition of meta-analyses, omitting one study in turn. The remaining clinicopathological variables (T status, N status, and clinical stage) were more sensitive, suffering further variations in their effect sizes, but under no circumstance shifted toward statistical significance.

#### 3.5.3. Analysis of Small-Study Effects

Visual inspection analysis of the asymmetry of the funnel plots constructed and the statistical tests conducted for the same purpose potentially indicated the absence of small-study effects (OS: *p*_Egger-TERT mutation_ = 0.376, *p*_Egger-TERT protein overexpression_ = 0.071; DFS: *p*_Egger-TERT mutation_ = 0.517; T status: *p*_Egger-TERT mutation_ = 0.493, *p*_Egger-TERT protein overexpression_ = 0.403; N status: *p*_Egger-TERT mutation_ = 0.338, *p*_Egger-TERT protein overexpression_ = 0.526; clinical stage: *p*_Egger-TERT protein overexpression_ = 0.546; histological grade: *p*_Egger-TERT mutation_ = 0.307, *p*_Egger-TERT protein overexpression_ = 0.349), although some analyses were performed under suboptimally underpowered conditions (n < 10 studies), where publication bias could not be strongly ruled out (Figures S12–S17, Supplementary Materials).

### 3.6. Validation of Methodological Quality

The methods applied in this systematic review and meta-analysis were implemented, critically appraised, and validated using AMSTAR2 [16], obtaining an overall rating of “high” (16 points) (the scoring table is included in the Supplementary Materials).

## 4. Discussion

The results of our systematic review and meta-analysis on 21 studies and 1698 patients with oral cancer demonstrate for the first time and based on evidence that TERT upregulation is predictive of the risk of death in patients with oral cancer, i.e., overall survival. This predictive capacity linked to TERT activity was statistically different depending on the type of analysis performed to detect its upregulation (*p* = 0.001), the immunohistochemical determination of TERT protein overexpression being the only efficient way to predict the risk of death from oral cancer (*p* < 0.001). Immunohistochemistry results showed an oral cancer mortality rate 3.01 times higher in patients who overexpressed TERT vs. those who did not. Regarding the influence of cancer hallmarks on the survival of oral cancer patients, the effect size related to the predictive value of overall survival derived from TERT immunohistochemical overexpression is one of the largest documented in evidence-based studies (systematic reviews and meta-analyses), together with metalloproteinase-2 overexpression [56]. It should be emphasized that TERT gene mutations did not predict the risk of death linked to TERT upregulation at all (*p* = 0.83, HR = 1.03), indicating that prognostic assessment of patients with oral cancer linked to TERT upregulation should be performed by immunohistochemistry of the protein in tumor tissue. TERT promoter-region mutations have been reported to confer different effects and are currently not fully understood. It has been hypothesized that increased TERT gene transcriptional activity creates binding motifs for E-twenty-six (ETS)/ternary complex factor (TCF) transcription factors, thereby allowing the synthesis of the catalytic subunit of telomerase TERT, determinant for telomerase activity and for cancer cell immortality [49]. On the other hand, alternative mechanisms have been published, such as the polymorphism rs2853669 T > C, which could disrupt a preexisting ETS binding site within the TERT core promoter, which on the contrary could result in decreased TERT protein expression [50]. Similar results to those discussed above were obtained for the disease-free survival parameter; that is, TERT upregulation was significantly associated with shorter disease-free survival (*p* = 0.006), and in the subgroup analysis, the highest predictive value was also achieved with the analysis of immunohistochemical overexpression of TERT in tumor tissue (*p* = 0.001, HR = 4.03). On the contrary, TERT gene mutations had no predictive effect on disease-free survival (*p* = 0.46, HR = 1.13). Moreover, although the analysis of TERT mRNA overexpression also predicted shorter disease-free survival (*p* = 0.05; HR = 3.79), the effect size was smaller than that obtained with immunohistochemistry and the results were also less robust, so as the available evidence indicates, it is more advisable to perform immunohistochemistry, a simple and automated technique. 

An interesting result of our meta-analysis is that the negative prognostic value of TERT upregulation in oral cancer showed no geographical differences (*p* = 0.27), which indicates in our view that the upregulation of this protein does not depend on etiological factors of oral cancer that act differently in different areas of the world, but probably represents a condition inherent to the biopathology of oral cancer. Furthermore, our results also indicate that the negative influence of TERT on survival is not affected by confounding factors that influence prognosis: follow-up period, sex, age, or alcohol/tobacco consumption. An additional planned subgroup meta-analysis stratified by anti-TERT antibody could not be performed, due to the heterogeneous and small amount of data reported by the primary-level studies. This is an important topic due to the possible aspecific reactivity of TERT antibodies that were used in immunohistochemical staining [40]. Therefore, the use of low-quality TERT antibodies may have contributed confusions in the conflicting reports of data on TERT protein, and undoubtedly impact on the conclusions of this manuscript. Likewise, a subgroup meta-analysis stratified by anatomical location could not be carried out, due to this information being reported by few of the studies included in our systematic review. This analysis would also have harbored important scientific value, because TERT promoter mutations have been found in higher relative frequencies in the mobile tongue than other oral subsites [49]. Future studies should further elucidate the potential influence of these parameters on our study conclusions.

The reasons that TERT upregulation negatively affects patient survival are linked to its canonical function, i.e., maintenance of telomere length with increased tumor cell survival [3]. However, in recent years, noncanonical functions of TERT performed outside the context of its telomeric actions [3] have also been reported. Animal experimentation has shown that ectopic expression of TERT promotes tumor formation and growth [57,58,59,60], and its constitutive overexpression increases the survival and replicative activity of tumor cells, even in the absence of growth factors, oxygen, or glucose [57,61,62], while conversely a lack of TERT activity promotes apoptosis [63,64,65,66,67,68]. Some of these noncanonical functions seem to be achieved by the ability of TERT to act as a transcriptional cofactor in the WNT/β-catenin pathway [22,69,70] and as a cofactor of NF-kβ, thus contributing to the regulation of the target genes of this transcription factor and as a modulator of MYC-dependent transcriptional programs involved in tumorigenesis [71]. TERT also possesses non-telomeric DNA repair activity [72], showing accelerated repair of nucleotide excision and double-strand DNA breaks [59,73,74]. In this regard, we also interestingly found that TERT upregulation was significantly associated with the development of poorly differentiated tumors (*p* < 0.001, OR = 3.20), and not with other parameters such as T, N, or clinical stage. In our view, this result indicates that the prosurvival function of TERT secondary to its telomere length-maintenance activity is probably predominant; thus, it is plausible to hypothesize that long-surviving malignant cells could, through a clonal mechanism, acquire summative advantages that would make them progressively more undifferentiated.

According to our qualitative evaluation, carried out using the QUIPS tool (developed by members of the Cochrane Prognosis Methods Group [75]), although the studies in our meta-analysis had similar experimental designs, not all were conducted with the same methodological rigor. Most potential biases were caused by the failure to consider potential confounding factors (i.e., domain 5: study-confounding domain) and by the application of incomplete or inappropriate statistical analyses (i.e., domain 6: statistical analysis and reporting). A comprehensive analysis of domain 5 helps to analyze if another factor may explain the study’s reported associations, a potential weakness often found in designs of an observational nature. In order to overcome this problem, we performed several meta-regression analyses. We could confirm that the main covariates (i.e., sex, age, follow-up period, tobacco and alcohol consumption) did not have an impact on the reported association, and none of them behaved as a confounding factor. Domain 6 addresses the appropriateness of the study’s statistical analysis and completeness of reporting. No primary-level studies directly reported effect-size metrics for survival analyses (i.e., hazard ratios with their corresponding 95% confidence intervals), which is unfortunately a common practice in studies of prognostic factors in cancer science. We overcome this potential weakness, we estimated hazard ratios from the data provided by these studies, following Parmar et al. [25] and Tierney et al. [24] adjustment methods. Future studies on the prognostic value of TERT upregulation in OSCC should consider the potential biases reported in this systematic review and meta-analysis, in order to improve and standardize future research.

Some potential limitations of our meta-analysis should be discussed. First, moderate heterogeneity was found between TERT upregulation and overall survival. Fortunately, the subgroup meta-analysis by alteration analyzed (i.e., gene mutation vs. protein overexpression) strongly demonstrated that heterogeneity was not significant after this stratification, showing homogeneous and well-balanced subgroups in terms of consistency (I^2^ = 0.0%, respectively). In summary, after an extensive exploratory analysis of sources of heterogeneity, we are seriously convinced that heterogeneity does not really constitute a concerning limitation of the present work. Second, publication bias could not be fully discarded, due to some variables showing fewer than 10 observations, making it impossible to differentiate between significant funnel-plot asymmetry from a chance-associated distribution. Therefore, although our analyses potentially ruled out small-study effects, publication bias is a real challenge currently omnipresent in biomedical science research [76]. Despite the above limitations, our study is robust, (i.e., reliable and stable, as confirmed by our meta-analytical primary results, stratifications, meta-regressions, sensitivity, and small-study effect analyses), presenting the first meta-analysis on this topic and reporting relevant and large effect size for TERT overexpression’s prognostic value in OSCC.

## 5. Conclusions

In conclusion, the immunohistochemical determination of TERT overexpression is an indicator of poor survival in oral cancer and probably should be incorporated in the prognostic evaluation of these patients. This meta-analysis also reinforces the need for further research on the potential applications of TERT as a therapeutic target in oral cancer.

## Figures and Tables

**Table 1:** Summary of the main characteristics of the study. Table S2 (Supplementary Materials) exhibits in detail the characteristics of each primary-level study included in this systematic review and meta-analysis.

Total Sample Size	21 Studies
Total patients (range)	1698 (30–218)
Year of publication	1999–2022
Study design	
Retrospective cohort	21 studies
TERT upregulation analysis	
Gene mutation	7 studies (959 patients)
mRNA expression	4 studies (174 patients)
protein overexpression	10 studies (565 patients)
Study continent	
Asia	8 studies (523 patients)
Europe	8 studies (769 patients)
North America	3 studies (361 patients)
South America	1 study (30 patients)

**Table 2:** Meta-analyses of prognostic and clinicopathological significance of TERT upregulation in OSCC.

					Pooled Data		Heterogeneity		
Meta-Analyses	Studies, n	Patients, n	Stat. Model	Wt	ES (95% CI)	*p*-Value	*P_het_*	*I^2^* (%)	Supplementary Materials ^a^
Overall Survival									
TERT upregulation (all) ^b^	9	1068	REM	D-L	HR = 1.40 (0.95–2.07)	0.09	0.02	55.0	Manuscript, Figure 3
Subgroup analysis by alteration ^c^						0.001 ^d^			
TERT mutations	6	892	REM	D-L	HR = 1.03 (0.80–1.31)	0.83	0.43	0.0	
TERT protein overexpression	3	176	REM	D-L	HR = 3.01 (1.70–5.35)	<0.001	0.48	0.0	
Subgroup analysis by geographical area ^c^						0.27 ^d^			Figure S1
Asian	3	336	REM	D-L	HR = 2.09 (0.86–5.08)	0.11	0.04	68.4	
Non-Asian	6	732	REM	D-L	HR = 1.19 (0.77–1.84)	0.43	0.11	45.0	
Univariable meta-regressions by study design and patients characteristics ^e^									
Follow-up (months)	9	1068	random-effects meta-regression		Coef = −0.002 (−0.010 to 0.006)	0.58 ± 0.005 ^f^	het_explained_ = −24.83%^g^		Figure S2
Sex (proportion of males, %)	8	1027	random-effects meta-regression		Coef = −0.005 (−0.052 to 0.043)	0.13 ± 0.003 ^f^	het_explained_ = −80.22% ^g^		Figure S3
Age (years, mean)	8	911	random-effects meta-regression		Coef = −0.003 (−0.122 to 0.115)	0.92 ± 0.003 ^f^	het_explained_ = -−43.25%^g^		Figure S4
Clinical stage (proportion of stage-III/IV patients,%)	2	295	—		—	—	—		—
Tobacco consumption (proportion of smokers, %)	7	974	random-effects meta-regression		Coef = 0.005 (−0.030 to 0.040)	0.71 ± 0.005 ^f^	het_explained_ = −74.01% ^g^		Figure S5
Areca nut/betel quid consumption (proportion of chewers, %)	2	283	—		—	—	—		—
Alcohol consumption (% of patients with positive habit)	6	817	random-effects meta-regression		Coef = 0.008 (−0.019 to 0.035)	0.44 ± 0.005 ^f^	het_explained_ = −41.06% ^g^		Figure S6
**Disease-free survival**									
TERT upregulation (all) ^b^	8	967	REM	D-L	HR = 1.64 (1.06–2.54)	0.03	0.07	46.4	Figure S7
Subgroup analysis by alteration ^c^						0.006 ^d^			
TERT mutations	5	790	REM	D-L	HR = 1.13 (0.81–1.59)	0.46	0.68	0.0	
TERT mRNA overexpression	1	42	—	—	HR = 3.79 (1.03–13.98)	0.05	—	0.0	
TERT protein overexpression	2	135	REM	D-L	HR = 4.03 (1.80–9.05)	0.001	0.49	0.0	
**T status**									
TERT upregulation (all) ^b^	11	1055	REM	D-L	OR = 1.15 (0.66–2.03)	0.62	0.001	65.4	Figure S8
Subgroup analysis by alteration ^c^						0.22 ^d^			
TERT mutations	4	569	REM	D-L	OR = 0.89 (0.56–1.40)	0.61	0.41	0.0	
TERT mRNA overexpression	1	42	—	—	OR = 0.40 (0.09–1.84)	0.24	—	0.0	
TERT protein overexpression	6	444	REM	D-L	OR = 1.81 (0.69–4.73)	0.23	0.001	77.3	
**N status**									
TERT upregulation (all) ^b^	10	1013	REM	D-L	OR = 1.25 (0.62–2.50)	0.54	<0.001	75.4	Figure S9
Subgroup analysis by alteration ^c^						0.21 ^d^			
TERT mutations	4	569	REM	D-L	OR = 0.80 (0.50–1.29)	0.36	0.78	0.0	
TERT protein overexpression	6	444	REM	D-L	OR = 1.82 (0.56–5.90)	0.32	<0.001	84.5	
**Clinical stage**									
TERT upregulation (all) ^b^	7	526	REM	D-L	OR = 1.33 (0.41–4.34)	0.64	<0.001	86.4	Figure S10
Subgroup analysis by alteration ^c^						0.24 ^d^			
TERT mutations	2	295	REM	D-L	OR = 0.66 (0.38–1.17)	0.16	0.88	0.0	
TERT mRNA overexpression	1	42	—	—	OR = 0.14 (0.01–2.64)	0.19	—	0.0	
TERT protein overexpression	4	189	REM	D-L	OR = 2.75 (0.34–22.61)	0.64	<0.001	91.6	
**Histological grade**									
TERT upregulation (all) ^b^	13	630	REM	D-L	OR = 1.94 (1.14–3.30)	0.01	0.21	23.0	Figure S11
Subgroup analysis by alteration ^c^						0.02 ^d^			
TERT mutations	1	144	—	—	OR = 0.42 (0.09–1.98)	0.28	—	0.0	
TERT mRNA overexpression	4	174	REM	D-L	OR = 1.16 (0.48–2.76)	0.74	0.44	0.0	
TERT protein overexpression	8	312	REM	D-L	OR = 3.20 (1.83–5.62)	<0.001	0.69	0.0	

Abbreviations: Stat., statistical; Wt, method of weighting; ES, effect-size estimation; HR, hazard ratio; OR, odds ratio; CI, confidence interval; REM, random-effects model; D-L, DerSimonian and Laird method; OSCC, oral squamous cell carcinoma. ^a^—More information in the Supplementary Materials, ^b^—Meta-analysis of aggregate (summary) data. ^c^—Subgroup meta-analyses, ^d^—Test for between-subgroup differences, ^e^—Meta-regression analysis of the potential effect of study covariates on the association between TERT upregulation and overall survival in OSCC. A meta-regression coefficient >0 indicates a greater impact of covariates on poor prognosis. ^f^—*p*-value ± standard error recalculated after 10,000 permutations based on Monte Carlo simulations. ^g^—Proportion of between-study variance explained (adjusted R^2^ statistic) using the residual maximum likelihood (REML) method. A negative number for proportion of heterogeneity explained reflects no heterogeneity explained.

## Data Availability

The data that support the findings of this study are available in the Supplementary Materials of this article.

## Supplementary Materials

The following supporting information can be downloaded at: https://www.mdpi.com/article/10.3390/cancers14153673/s1, Figures S1–S30, Tables S1–S3, List of excluded and included studies.
