# Supplementary material for: Prognostic and Clinicopathological Significance of Telomerase Reverse Transcriptase Upregulation in Oral Cancer: A Systematic Review and Meta-Analysis
Source: Cancers (Basel). 2022 Jul 28;14(15):3673. doi: 10.3390/cancers14153673 (PMC9367569; doi:10.3390/cancers14153673)
Supplement: Supplementary file 1 [file cancers-14-03673-s001.zip › cancers-1805929-supplementary.pdf]

cancers

MDPI

Supplementary material

Prognostic and Clinicopathological Significance of Telomerase Reverse Transcriptase Upregulation in Oral Cancer: A Systematic Review and Meta-Analysis

Miguel Ángel González-Moles, Eloísa Moya-González, Alberto García-Ferrera, Paola Nieto-Casado, and Pablo Ramos-García

Table of contents

1. Search strategy. Table S1. 3
2. Characteristics of analyzed studies. Table S2 4
3. Meta-analysis on the association between TERT upregulation and OS in OSCC 6
    3.1 Subgroup meta-analysis by geographical area 6
    3.2 Univariable meta-regression on the effect of follow up 7
    3.3 Univariable meta-regression on the effect of sex 8
    3.4 Univariable meta-regression on the effect of age 9
    3.5 Univariable meta-regression on the effect of tobacco 10
    3.6 Univariable meta-regression on the effect of alcohol 11
4. Meta-analysis on the association between TERT upregulation and DFS 12
5. Meta-analysis on the association between TERT upregulation and T status 13
6. Meta-analysis on the association between TERT upregulation and N status 14
7. Meta-analysis on the association between TERT upregulation and clinical stage 15
8. Meta-analysis on the association between TERT upregulation and histol.grade 16
9. Analysis of small-study effects. 17
    9.1 TERT upregulation and overall survival in OSCC 17
    9.2 TERT upregulation and DFS in OSCC 18
    9.3 TERT upregulation and T status in OSCC 19
    9.4 TERT upregulation and N status in OSCC 20
    9.5 TERT upregulation and clinical stage in OSCC 21
    9.6 TERT upregulation and histological grade in OSCC 22
10. Sensitivity analysis ("leave-one-out method"). 23
    10.1 TERT upregulation and overall survival in OSCC 23
    10.2 TERT upregulation and DFS in OSCC 24
    10.3 TERT upregulation and T status in OSCC 25
    10.4 TERT upregulation and N status in OSCC 26

10.5 TERT upregulation and clinical stage in OSCC 27
10.6 TERT upregulation and histological grade in OSCC 28
11. Analysis of secondary clinico-pathological variables. 29
12. List of excluded studies with reasons. 30
13. Validation of methodological quality 33

1. Search strategy

Table S1. Search strategy for each database, number of results, and execution date.

Database	Query/Search Strategy	Results/ Items found	Search time limits
PubMed	("Telomerase"[Mesh] OR "telomerase"[All Fields] OR "tert"[All Fields] OR "htert"[All Fields] OR "trt"[All Fields] OR "tp2"[All Fields] OR "tcs1"[All Fields] OR "hest2"[All Fields] OR "est2"[All Fields] OR "telomerase reverse transcriptase"[All Fields]) AND ("mouth"[MeSH Terms] OR "mouth"[All Fields] OR "oral"[All Fields] OR oropharyn*[All Fields]) AND ("carcinoma, squamous cell"[MeSH Terms] OR ("carcinoma"[All Fields] AND "squamous"[All Fields] AND "cell"[All Fields]) OR "squamous cell carcinoma"[All Fields] OR "Neoplasms"[Mesh Terms] OR neopla*[All Fields] OR "cancer"[All Fields] OR "dysplasia"[All Fields] OR "potentially malignant disorders"[All Fields] OR premalign*[All Fields] OR precancer*[All Fields] OR "leukoplakia"[All Fields] OR "erythroplakia"[All Fields] OR "lichen planus"[All Fields] OR "submucous fibrosis"[All Fields] OR "malignant transformation"[All Fields])	524	April, 2022
Embase	('telomerase'/exp OR 'telomerase' OR 'tert' OR 'htert' OR 'trt' OR 'tp2' OR 'tcs1' OR 'hest2' OR 'est2' OR 'telomerase reverse transcriptase') AND ('mouth'/exp OR 'mouth' OR 'oral') AND ('squamous cell carcinoma'/exp OR 'carcinoma' OR 'malignant neoplasm'/exp OR 'neoplas*' OR 'cancer')	2161	April, 2022
Web of Science	TS=("telomerase" OR "tert" OR "htert" OR "trt" OR "tp2" OR "tcs1" OR "hest2" OR "est2" OR "telomerase reverse transcriptase") AND TS=("mouth" OR "oral") AND TS=("squamous cell carcinoma" OR neoplas* or "cancer")	1255	April, 2022
Scopus	TITLE-ABS-KEY(("telomerase" OR "tert" OR "htert" OR "trt" OR "tp2" OR "tcs1" OR "hest2" OR "est2" OR "telomerase reverse transcriptase") AND ("mouth" OR "oral") AND ("squamous cell carcinoma" OR neoplas* or "cancer"))	792	April, 2022
Total	4732		

Table S2. Characteristics of analyzed studies (n = 21)

Study	Year	Country	Publ. language	Study design	Recruitment period	Follow up, m,mean±SD (range)	Sample Size, n	Sex M, n (%) F, n (%)	Age, y mean±SD (range)	Tumour subsites									
Haraguchi *et al*	2022	Japan	English	Retrospective cohort	2013–2015	NR (0-60)	53	M, 28 (52,83) F, 25 (47,17)	64,5±NR (33-93)	Tongue, gingiva, buccal mucosa	NR	NR	Protein	IHQ	Clone 2C4 (monoclonal)	1:40 NR NR	nuclear	50	27 (50.94)
Moreira *et al.*	2021	France	English	Retrospective cohort	1991–2016	42±NR (1–258)	151	M, 92 (60.93) F, 59 (39.07)	64 ±NR (23–91)	NR	+: 73 (48.34) -: 65 (43.05)	+:48(31.79) -:81 (53.64)	Mutation	NGS	NA	NA	NA	NA	76 (50.33)
Yu *et al*	2021	USA	English	Retrospective cohort	2004–2017	NR (0–72)	67	M, 42(62.69) F, 25(37.31)	55.87±13.3 8 (28-89)	Oral cavity	39 (58.21)	NR	Mutation	NGS	NA	NA	NA	NA	55 (82.09)
Giunco *et al.*	2021	Italy	English	Retrospective cohort	2010–2018	43 ± NR (28–75)	144	M: 81 (56.2) F: 63 (43.8)	65 ± NR (54–74)	Tongue, FOM, Gingiva, Buccal mucosa, Lip , Palate, RMT	+: 89 (61.8) -: 55 (38.19)	+:60 (41.7) -:84 (58.3)	Mutation	NGS	NA	NA	NA	NA	16 (11.1)
Yilmaz *et al.*	2020	Turkey	English	Retrospective cohort	NR	NR (0–141.10)	102	M, 58 (56.82) F, 44 (43.14)	61.70±14.4 7	Tongue , Palate, Gingiva, FOM, Buccal mucosa, RMT	+: 46 (45.10) -: 49 (48.04 Missing: 7 (6.86)	+:20(19.61) -: 75(73.53) Missing: 7 (6.86)	Mutation	NGS	NA	NA	NA	NA	77 (75.49)
Raju *et al*.	2019	India	English	Retrospective cohort	2015–2018	NR	20	M, 10 (50) F, 10 (50)	49.60 ± 13.08	Gingiva, Tongue, Buccal mucosa	NR	NR	Protein	IHQ	NCL (monoclonal)	1:25 overnight 4ºC	Nuclear and citoplas mic	NA (labeling score= intensity x cell count)	NR
Mishra *et al.*	2019	India	English	Retrospective cohort	NR	NR	15	M, 15 (100) F, 0 (0)	52.00 ± 6.40	NR	NR	NR	Protein	IHQ	NR (polyclonal)	NR 1h Room temperature	Nuclear Citoplas mic Both	NA (labeling score= intensity x cell count)	NR
Mundi *et al.*	2019	Canada	English	Retrospective cohort	2011–2015	32±NR (0–95.48)	137	M, 99 (72.26) F, 38 (27.74)	62.3±11.1 NR	NR	+: 98 (71.53) -: 39 (28.47)	+:54 (40) -: 81(60) Missing: 2	Mutation	NGS	NA	NA	NA	NA	42 (30,4)
Dogan *et al*	2019	USA	English	Retrospective cohort	1978–2005	67±NR (0.2–291)	157	M, 114 (72.61) F, 43 (27.39)	59.55±10.4 6 (27–84)	Tongue, Tonsil, Soft palate	129 (82.17)	NR	Mutation	NGS	NA	NA	NA	NA	9 (5.73)
Haraguchi *et al*	2017	Japan	English	Restrospective		NR	35	M, 20 (57.14)		Tongue, Gingiva									

				cohort	2013–2017			F, 15 (42.86)	64.23±14.37 (33–93)		NR	NR	Protein	IHQ	Clone 2C4 (monoclonal)	1:40<br>Overnight<br>4ºC	Nuclear	NA(labelling score=intensity x cell count)	NR
Chang *et al.*	2017	Taiwan	English	Retrospective cohort	2002–2007	NR	201	M: 182 (90.55)<br>F: 19 (9.45)	52.8 ± 11.3 (30.3-86.8)	Buccal mucosa, Gingiva, Lips, FOM, Tongue	Betel chewers:<br>+:156(77.61)<br>-: 45(22.39)<br>Smokers:<br>+:167 (83.08)<br>-:34 (16.92)	+:150 (74.63)<br>-: 51 (25.37)	Mutation	NGS	NA	NA	NA	NA	104 (51.7)
Zhao *et al.*	2015	China	English	Retrospective cohort	NR	NR	37	M: 24 (64.86)<br>F: 13 (35.13)	NR	Buccal mucosa,Others	NR	NR	Protein	IHQ	NR (polyclonal)	1:25<br>Overnight<br>4ºC	Cytoplasmic and nuclear	NA (labeling score = intensity x cell count)	NR
Abrahao *et al.*	2011	Brazil	English	Retrospective cohort	NR	NR	30	M:20 (66.67)<br>F: 10 (33.33)	NR, 62.17±NR (NR)	Tongue, Buccal mucosa, FOM, Gingiva, Palate	NR	NR	Protein	IHQ	Clone 44F12 (monoclonal)	1:75<br>30 minutes<br>Room temperature	Nuclear	NR	27(90)
Fabricius *et al.*	2009	Germany	English	Retrospective cohort	1994–1997	NR (0–151.32)	34	M: 30 (88.24)<br>F: 4 (11.76)	57.82 ± 8.65 (39–81)	FOM, Tongue, Gingiva, Tonsil, Palate, Buccal mucosa, Lips	NR	NR	Protein	IHQ	Ab177 (Polyclonal)	1:250<br>60 minutes drying oven 37º<br>30 minutes room temperature	Nuclear-cytoplasmic	NA (labeling score = intensity x cell count)	16 (47.06)
Chen *et al.*	2007	Taiwan	English	Retrospective cohort	1995–2002	NR (0–106.72)	82	M, 64 (78.05)<br>F, 18 (21.95)	51± NR (26–77)	buccal mucosa, tongue, gingiva, palate, lip, FOM	+: 61(74.39)<br>-: 21(25.61)	53 (64.63)	protein	IHQ	Clone 44F12 (monoclonal)	1:50<br>Overnight<br>4ºC	Nuclear	NA (labeling score= intensity x cell count)	NR
Panone *et al.*	2007	Italy	English	Retrospective cohort	NR	NR (36–53.22)	41	NR	65± NR (42–81)	Tongue, Gingiva, Palate, RMT, FOM	NR	NR	protein	IHQ	EST22-A (polyclonal)	1:250<br>120<br>24ºC	Nuclear<br>Cytoplasmic<br>Both	30	16<br>(39.02)
Freier *et al.*	2007	Germany	English	Retrospective cohort	NR	NR	218	NR	60±NR (16–92)	NR	NR	NR	Protein	IHQ	NR<br>(polyclonal)	1:100<br>NR<br>NR	Nuclear	5	177 (81.19)
Zhou *et al*	2006	China	Chinese	Retrospective cohort	2001–2006	NR	65	NR	NR	NR	NR	NR	mRNA	RT-PCR	NA	NA	NA	NA	54 (83.1)
Luzar *et al*	2004	Slovenia	English	Retrospective cohort	1998–2001	NR	37	NR	NR	NR	NR	NR	mRNA	RT-PCR	NA	NA	NA	NA	25 (68)

								NR											
Lee *et al*	2001	Germany	English	Retrospective cohort	NR	NR<br>(3-60)	42	NR<br>NR	NR	NR	NR	NR	mRNA	RT-PCR	NA	NA	NA	NA	34<br>(80.95)
Zhang *et al*.	1999	China	English	Retrospective cohort	1997–1998	NR	30	NR	NR	NR	NR	NR	mRNA	ISH	NR	NR	Citoplas<br>mic	NR	24<br>()

Abbreviations: Bm, buccal mucosa; fom, floor of mouth; OSCC, oral squamous cell carcinoma; m, months; n, number; NR, not reported; SD, standard deviation; y, years, NGS, next generation sequencing; IHQ, immunohistochemistry, RT-PCR, real time polymerase chain reaction; ISH, in situ hybridization; TERT, telomerase reverse transcriptase.

3. Meta-analysis on the association between TERT upregulation and OS in OSCC

3.1. Subgroup meta-analysis by geographical area

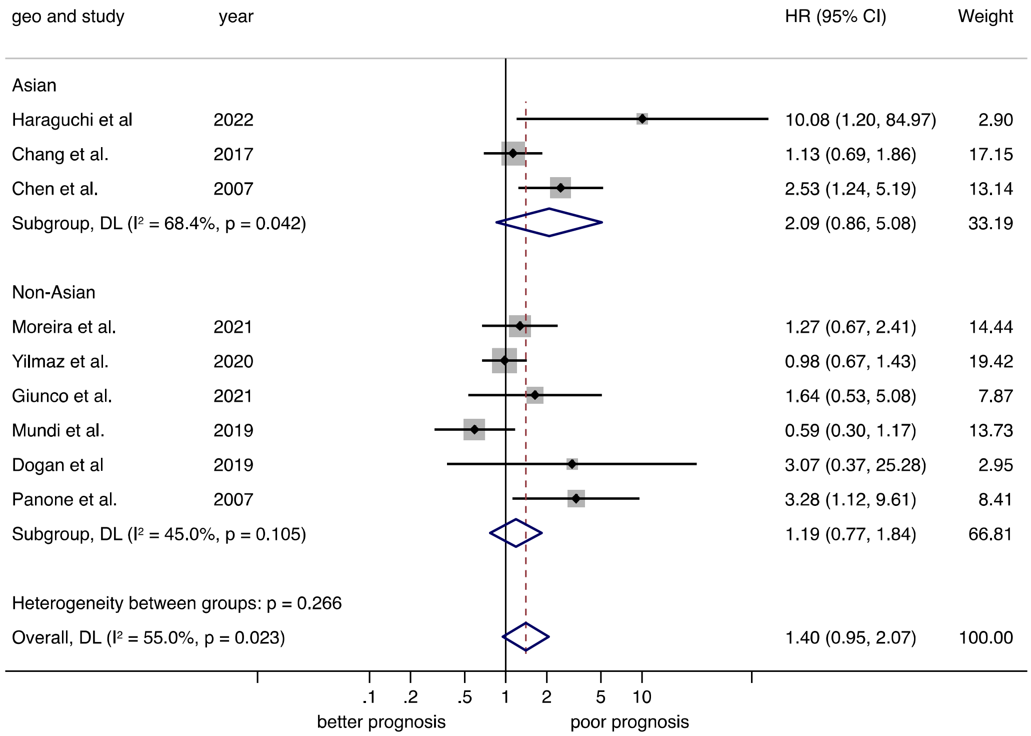

Figure S1. Forest plot graphically representing the subgroup meta-analysis by geographical area (Asian vs. Non-Asian) of the association between TERT upregulation and overall survival in patients with OSCC. TERT, telomerase reverse transcriptase; OSCC, oral squamous cell carcinoma; HR, hazard ratio; CI, confidence intervals. Random-effects model, inverse-variance weighting (based on the DerSimonian and Laird method). A HR > 1 suggests that TERT upregulation is associated with poor prognosis. Diamonds indicate the pooled HRs with their corresponding 95% CIs.

3.2. Univariable meta-regression on the effect of follow up

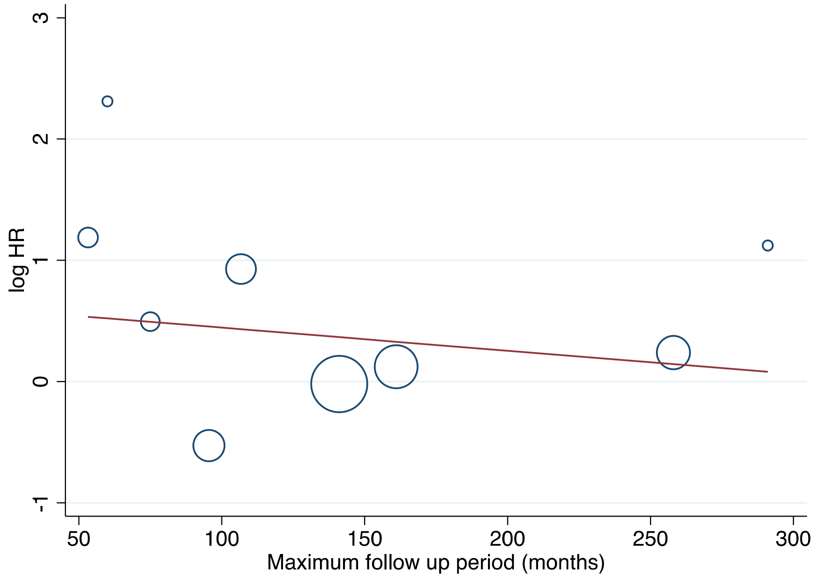

Figure S2. Bubble plot graphically representing the univariable meta-regression analysis of the potential effect of follow up period (expressed in months) on the association between TERT upregulation and overall survival in patients with OSCC. TERT, telomerase reverse transcriptase; OSCC, oral squamous cell carcinoma; HR, hazard ratio; log, natural logarithm (i.e., log base e). The red line exhibits the fitted regression line together with blue circles representing the estimates from each individual study, sized according to the precision of each estimate (the inverse of its within-study variance).

3.3. Univariable meta-regression on the effect of sex

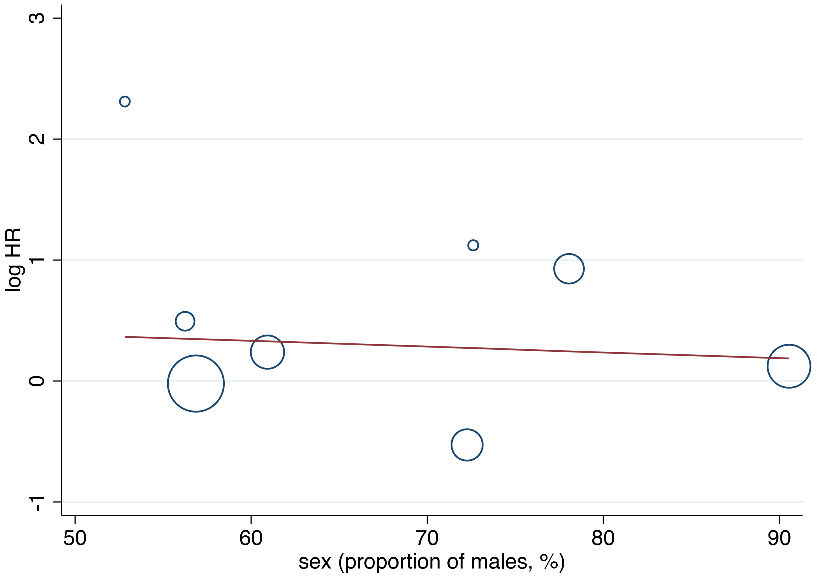

Figure S3. Bubble plot graphically representing the univariable meta-regression analysis of the potential effect of sex (% of males) on the association between TERT upregulation and overall survival in patients with OSCC. TERT, telomerase reverse transcriptase; OSCC, oral squa-

mous cell carcinoma; HR, hazard ratio; log, natural logarithm (i.e., log base e). The red line exhibits the fitted regression line together with blue circles representing the estimates from each individual study, sized according to the precision of each estimate (the inverse of its within-study variance).

3.4. Univariable meta-regression on the effect of age

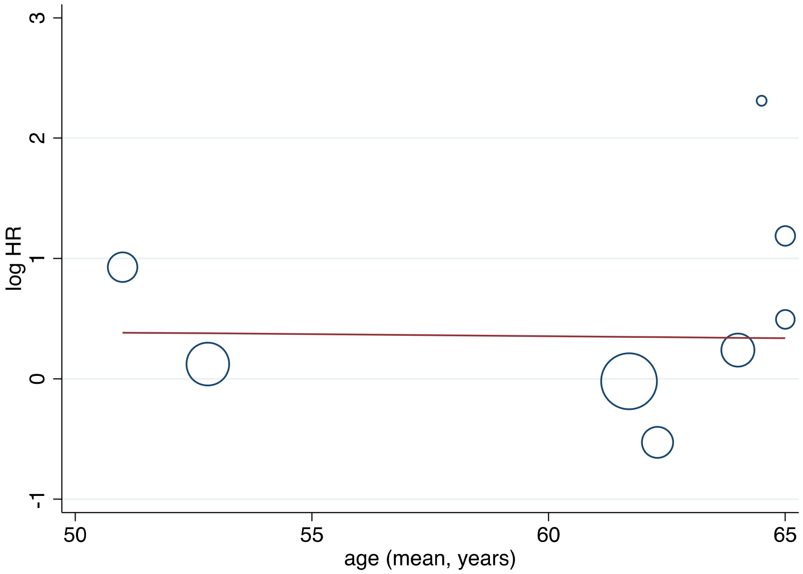

Figure S4. Bubble plot graphically representing the univariable meta-regression analysis of the potential effect of age (mean age of patients, expressed in years) on the association between TERT upregulation and overall survival in patients with OSCC. TERT, telomerase reverse transcriptase; OSCC, oral squamous cell carcinoma; HR, hazard ratio; log, natural logarithm (i.e., log base e). The red line exhibits the fitted regression line together with blue circles representing the estimates from each individual study, sized according to the precision of each estimate (the inverse of its within-study variance).

3.5. Univariable meta-regression on the effect of tobacco

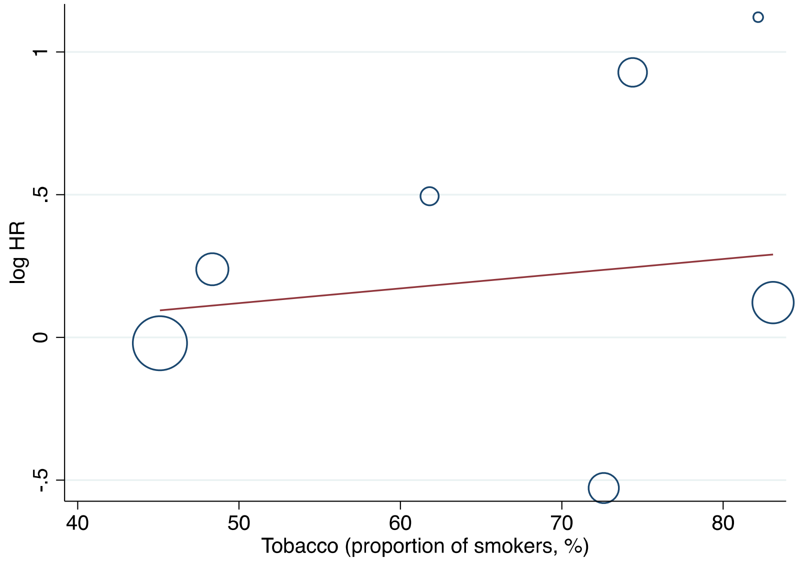

Figure S5. Bubble plot graphically representing the univariable meta-regression analysis of the potential effect of tobacco (% of smokers) on the association between TERT upregulation and overall survival in patients with OSCC. TERT, telomerase reverse transcriptase; OSCC, oral squamous cell carcinoma; HR, hazard ratio; log, natural logarithm (i.e., log base e). The red line exhibits the fitted regression line together with blue circles representing the estimates from each individual study, sized according to the precision of each estimate (the inverse of its within-study variance).

3.6. Univariable meta-regression on the effect of alcohol

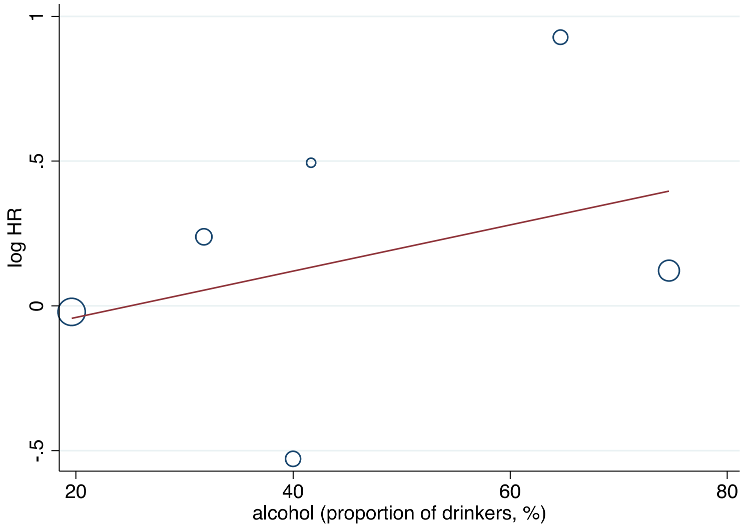

Figure S6. Bubble plot graphically representing the univariable meta-regression analysis of the potential alcohol (% of drinkers) on the association between TERT upregulation and overall survival in patients with OSCC. TERT, telomerase reverse transcriptase; OSCC, oral squamous cell carcinoma; HR, hazard ratio; log, natural logarithm (i.e., log base e). The red line exhibits the fitted regression line together with blue circles rep-

resenting the estimates from each individual study, sized according to the precision of each estimate (the inverse of its within-study variance).

4. Meta-analysis on the association between TERT upregulation and DFS

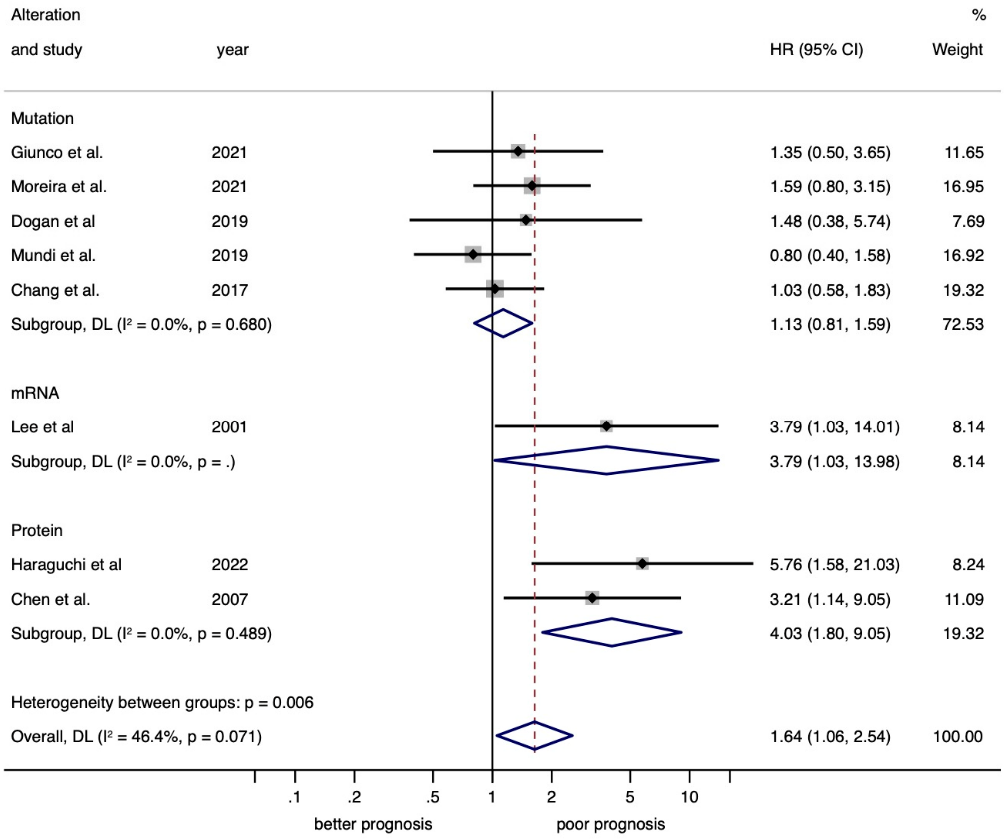

Figure S7. Forest plot graphically representing the meta-analysis on the association between TERT upregulation (stratified by alterations: mutations vs mRNA vs protein overexpression) and DFS in patients with OSCC. TERT, telomerase reverse transcriptase; DFS, disease-free survival; OSCC, oral squamous cell carcinoma; HR, hazard ratio; CI, confidence intervals. Random-effects model, inverse-variance weighting (based on the DerSimonian and Laird method). A HR > 1 suggests that TERT upregulation is associated with poor prognosis. Diamonds indicate the pooled HRs with their corresponding 95% CIs.

5. Meta-analysis on the association between TERT upregulation and T status

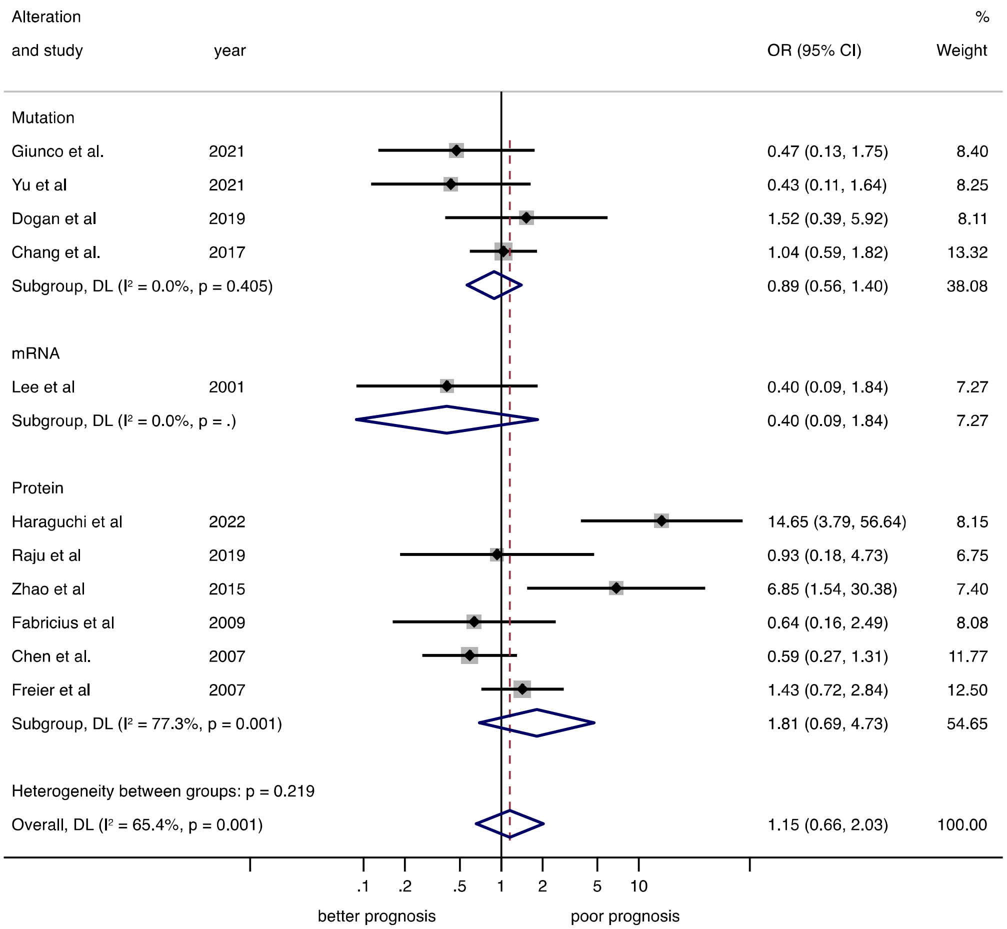

Figure S8. Forest plot graphically representing the meta-analysis on the association between TERT upregulation (stratified by alterations: mutations vs mRNA vs protein overexpression) and T status (T3/T4 vs. T1/T2) in patients with OSCC. TERT, telomerase reverse transcriptase; OSCC, oral squamous cell carcinoma; OR, odds ratio; CI, confidence intervals. Random-effects model, inverse-variance weighting (based on the DerSimonian and Laird method). A OR > 1 suggests that TERT upregulation is associated with poor prognosis. Diamonds indicate the pooled ORs with their corresponding 95% CIs.

6. Meta-analysis on the association between TERT upregulation and N status

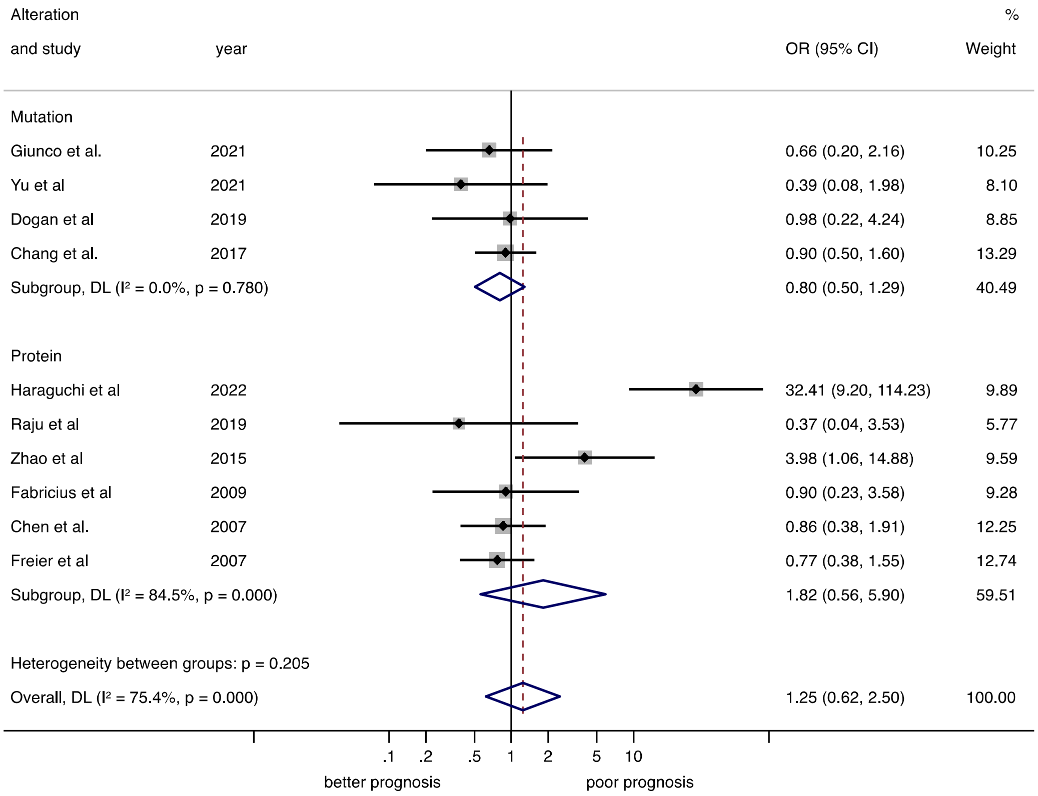

Figure S9. Forest plot graphically representing the meta-analysis on the association between TERT upregulation (stratified by alterations: mutations vs protein overexpression) and N status (positive metastatic lymph nodes vs. negative) in patients with OSCC. TERT, telomerase reverse transcriptase; OSCC, oral squamous cell carcinoma; OR, odds ratio; CI, confidence intervals. Random-effects model, inverse-variance weighting (based on the DerSimonian and Laird method). A OR > 1 suggests that TERT upregulation is associated with poor prognosis. Diamonds indicate the pooled ORs with their corresponding 95% CIs.

7. Meta-analysis on the association between TERT upregulation and clinical stage

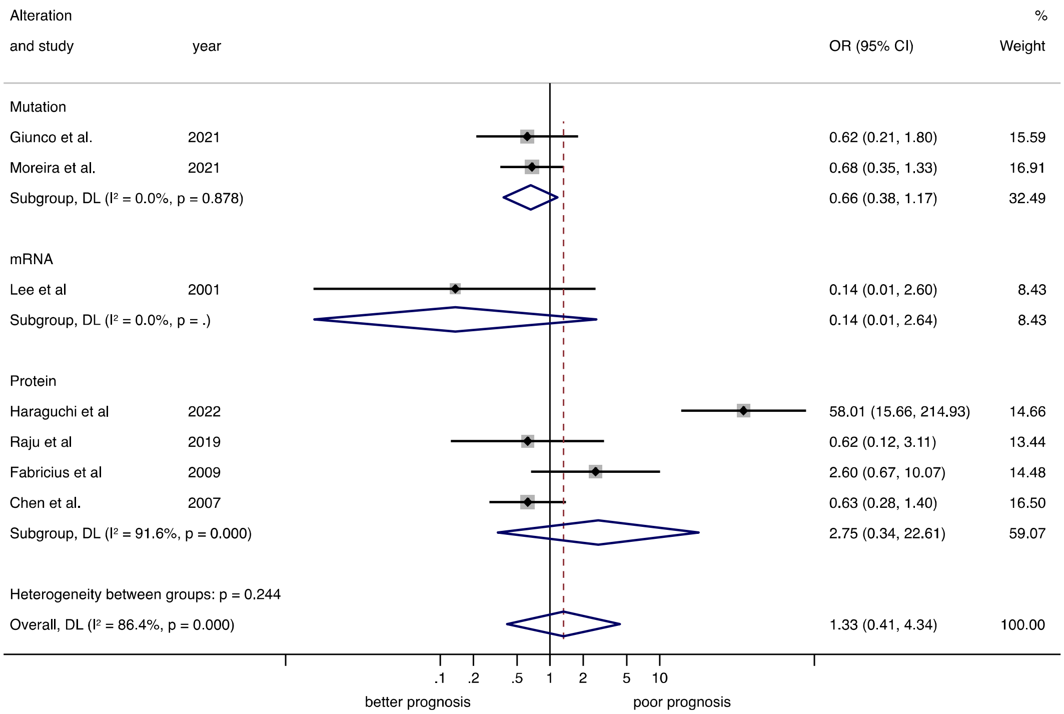

Figure S10. Forest plot graphically representing the meta-analysis on the association between TERT upregulation (stratified by alterations: mutations vs mRNA vs protein overexpression)and clinical stage (III/IV vs. I/II) in patients with OSCC. TERT, telomerase reverse transcriptase; OSCC, oral squamous cell carcinoma; OR, odds ratio; CI, confidence intervals. Random-effects model, inverse-variance weighting (based on the DerSimonian and Laird method). A OR > 1 suggests that TERT upregulation is associated with poor prognosis. Diamonds indicate the pooled ORs with their corresponding 95% CIs.

8. Meta-analysis on the association between TERT upregulation and histological grade

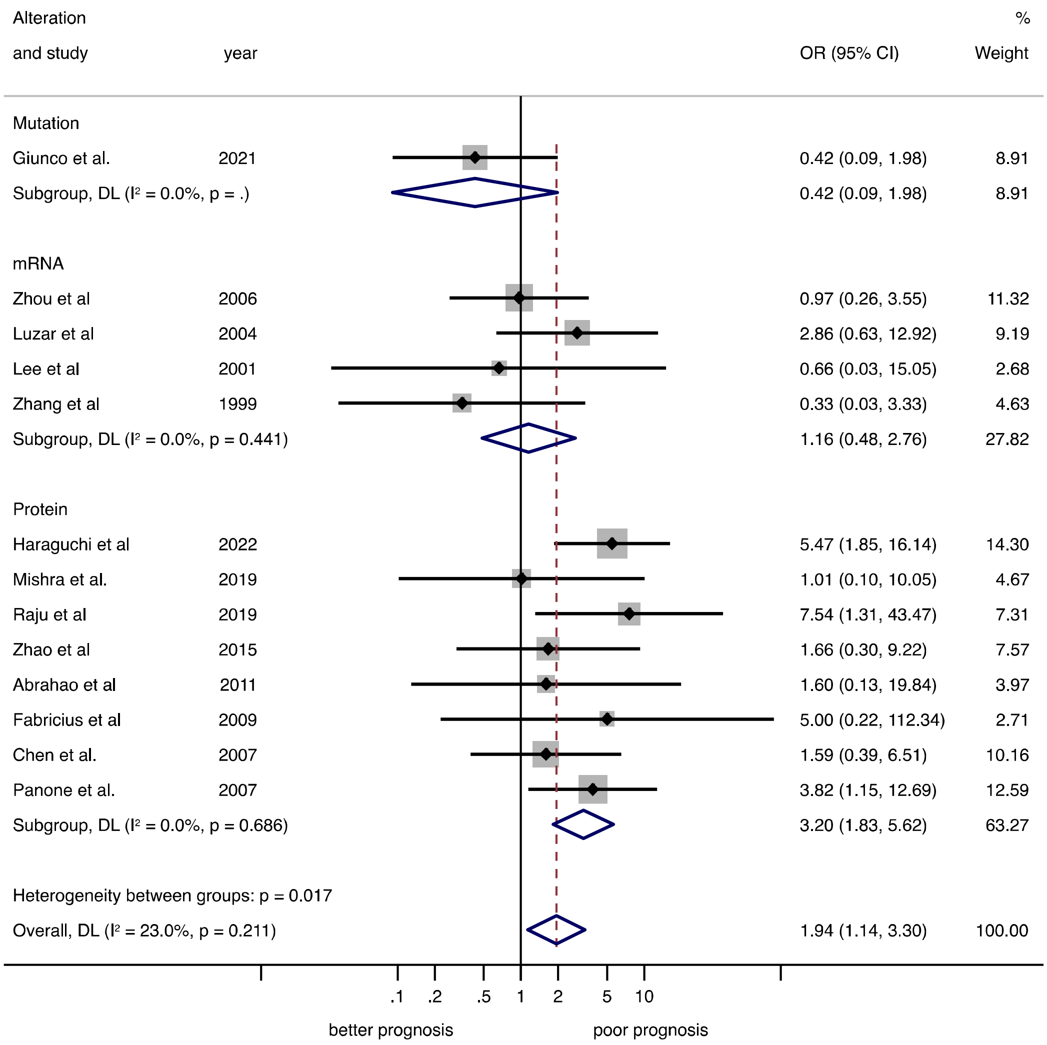

Figure S11. Forest plot graphically representing the meta-analysis on the association between TERT upregulation (stratified by alterations: mutations vs mRNA vs protein overexpression)n and histological grade (poorly-moderate vs. well-differentiated carcinomas) in patients with OSCC. TERT, telomerase reverse transcriptase; OSCC, oral squamous cell carcinoma; OR, odds ratio; CI, confidence intervals. Random-effects model, inverse-variance weighting (based on the DerSimonian and Laird method). A OR > 1 suggests that TERT upregulation is associated with poor prognosis. Diamonds indicate the pooled ORs with their corresponding 95% CIs.

9. Analysis of small-study effects

9.1. TERT upregulation and overall survival in OSCC

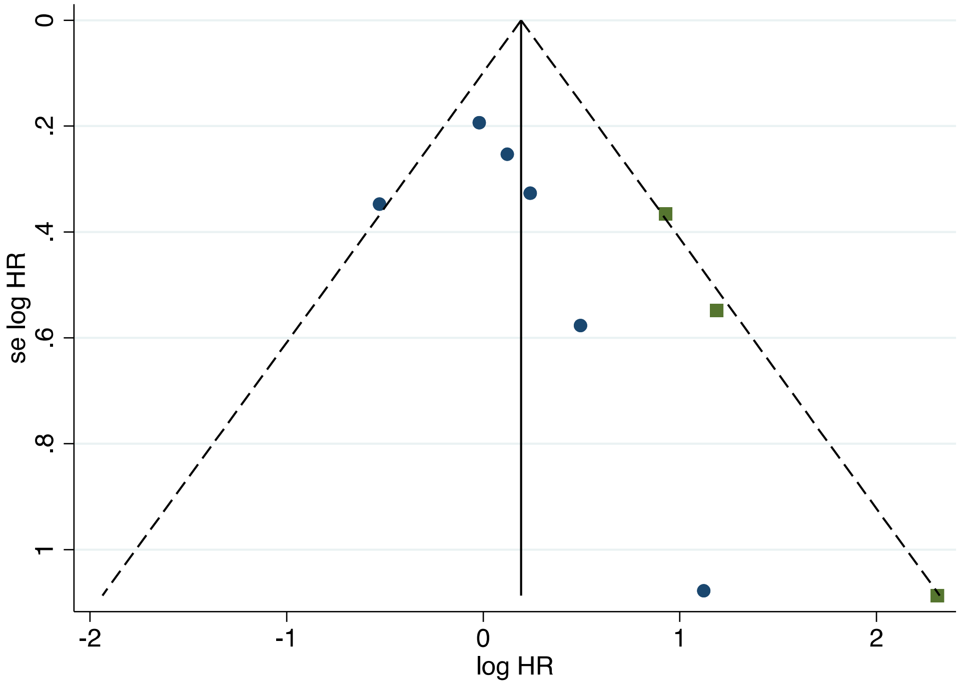

Figure S12. A funnel plot of estimated logHRs against their standard errors, graphically representing the analysis of small-study effects on the association between TERT upregulation and overall survival in OSCC. TERT, telomerase reverse transcriptase; OSCC, oral squamous cell carcinoma; SE, standard error; HR, hazard ratio; log, natural logarithm (i.e., log base e). The black vertical line corresponds to the pooled estimated prevalence. The two diagonal intermittent lines represent the pseudo-95% confidence interval. The blue circles (TERT mutations), and green squares (protein overexpression) represent the estimates from primary-level studies.

9.2. TERT upregulation and DFS in OSCC

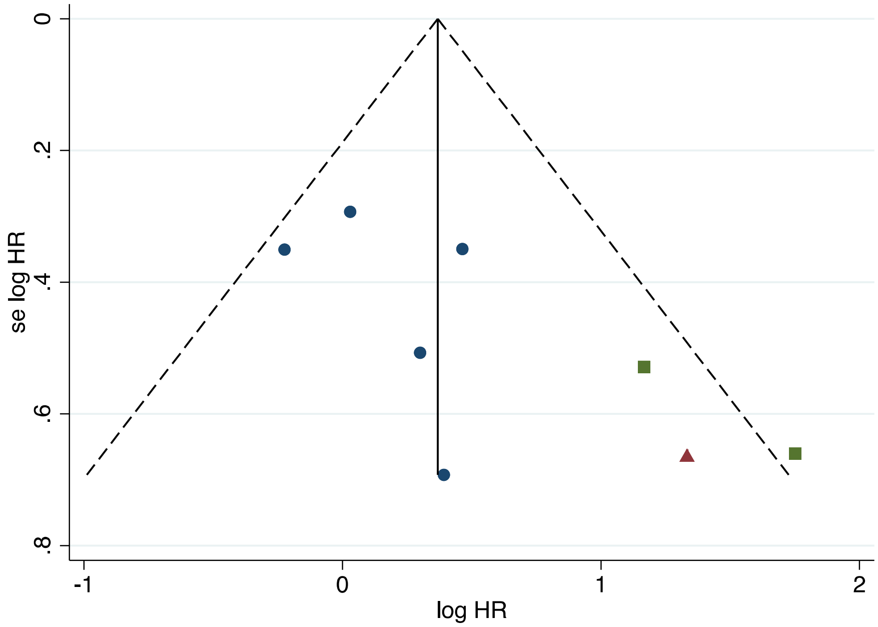

Figure S13. A funnel plot of estimated logHRs against their standard errors, graphically representing the analysis of small-study effects on the association between TERT upregulation and DFS in OSCC. TERT, telomerase reverse transcriptase; OSCC, oral squamous cell carcinoma; DFS, disease-free survival; SE, standard error; HR, hazard ratio; log, natural logarithm (i.e., log base e). The black vertical line corresponds to the pooled estimated prevalence. The two diagonal intermittent lines represent the pseudo-95% confidence interval. The blue circles (TERT mutations), green squares (protein overexpression) and red triangles (mRNA overexpression) represent the estimates from primary-level studies.

9.3. TERT upregulation and T status in OSCC

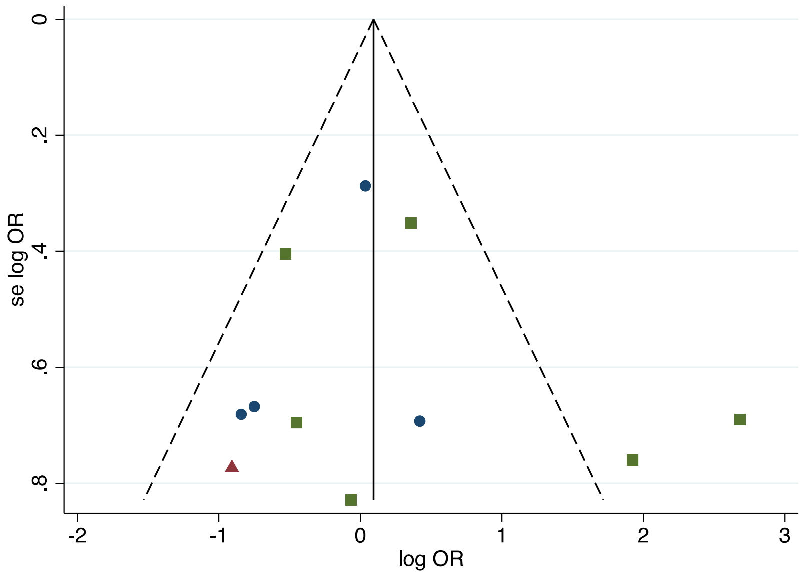

Figure S14. A funnel plot of estimated logORs against their standard errors, graphically representing the analysis of small-study effects on the association between TERT upregulation and T status in OSCC. TERT, telomerase reverse transcriptase; OSCC, oral squamous cell carcinoma; SE, standard error; OR, odds ratio; log, natural logarithm (i.e., log base e). The black vertical line corresponds to the pooled estimated prevalence. The two diagonal intermittent lines represent the pseudo-95% confidence interval. The blue circles (TERT mutations), green squares (protein overexpression) and red triangles (mRNA overexpression) represent the estimates from primary-level studies.

9.4. TERT upregulation and N status in OSCC

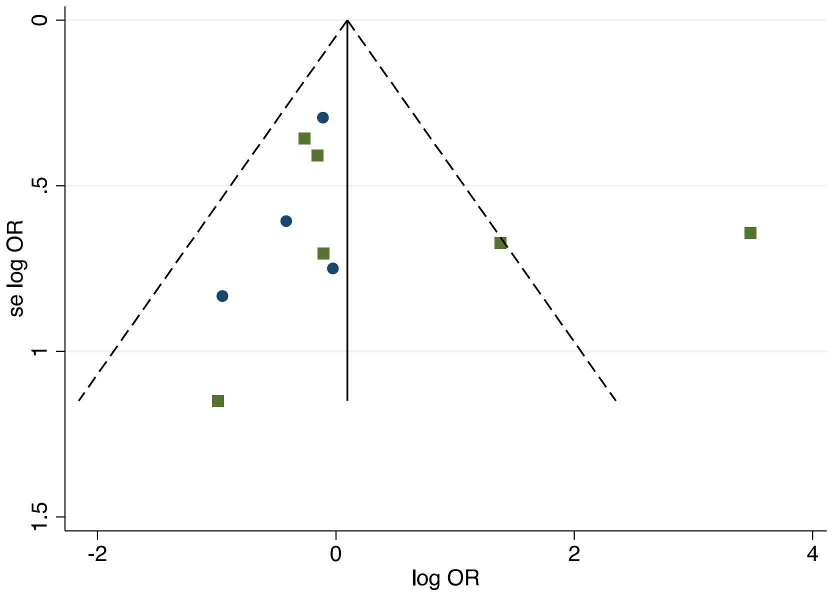

Figure S15. A funnel plot of estimated logORs against their standard errors, graphically representing the analysis of small-study effects on the association between TERT upregulation and N status in OSCC. TERT, telomerase reverse transcriptase; OSCC, oral squamous cell carcinoma; SE, standard error; OR, odds ratio; log, natural logarithm (i.e., log base e). The black vertical line corresponds to the pooled estimated prevalence. The two diagonal intermittent lines represent the pseudo-95% confidence interval. The blue circles (TERT mutations), green squares (protein overexpression) and green squares (protein overexpression) represent the estimates from primary-level studies.

9.5. TERT upregulation and clinical stage in OSCC

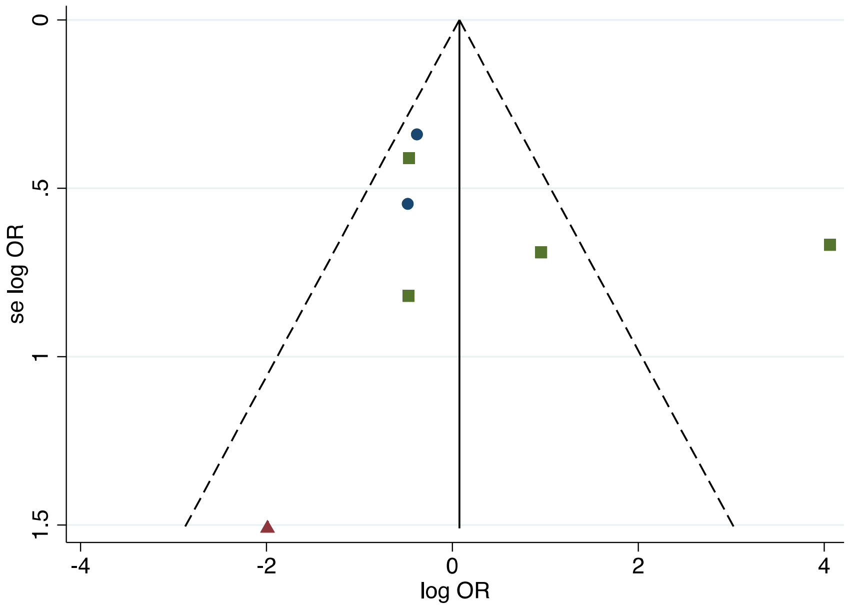

Figure S16. A funnel plot of estimated logORs against their standard errors, graphically representing the analysis of small-study effects on the association between TERT upregulation and clinical stage in OSCC. TERT, telomerase reverse transcriptase; OSCC, oral squamous cell carcinoma; SE, standard error; OR, odds ratio; log, natural logarithm (i.e., log base e). The black vertical line corresponds to the pooled estimated prevalence. The two diagonal intermittent lines represent the pseudo-95% confidence interval. The blue circles (TERT mutations), green squares (protein overexpression) and red triangles (mRNA overxpression) represent the estimates from primary-level studies.

9.6. TERT upregulation and histological grade in OSCC

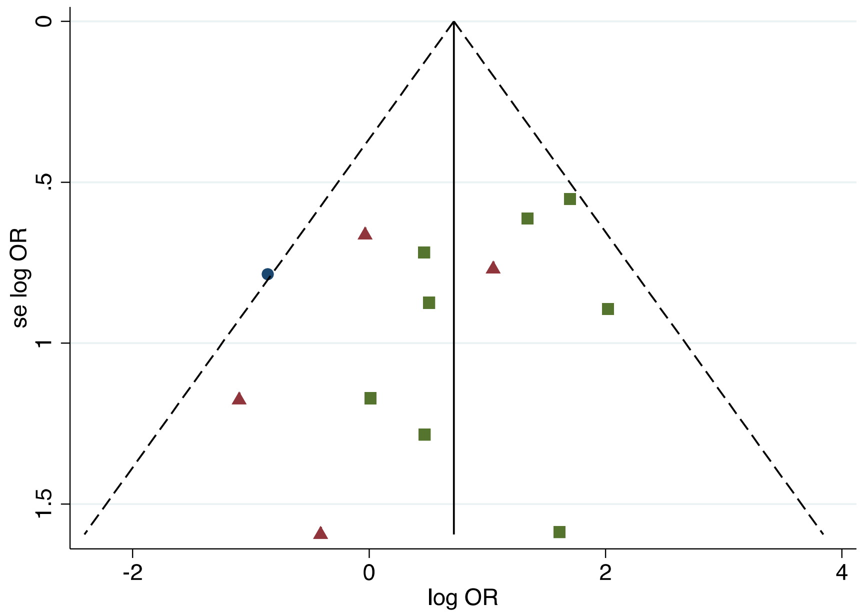

Figure S17. A funnel plot of estimated logORs against their standard errors, graphically representing the analysis of small-study effects on the association TERT upregulation and histological grade in OSCC. TERT, telomerase reverse transcriptase; OSCC, oral squamous cell carcinoma; SE, standard error; OR, odds ratio; log, natural logarithm (i.e., log base e). The black vertical line corresponds to the pooled estimated prevalence. The two diagonal intermittent lines represent the pseudo-95% confidence interval. The blue circles (TERT mutations), green squares (protein overexpression) and red triangles (mRNA overexpression) represent the estimates from primary-level studies.

10. Sensitivity analysis (leave-one-out method).

10.1. TERT upregulation and Overall Survival in OSCC

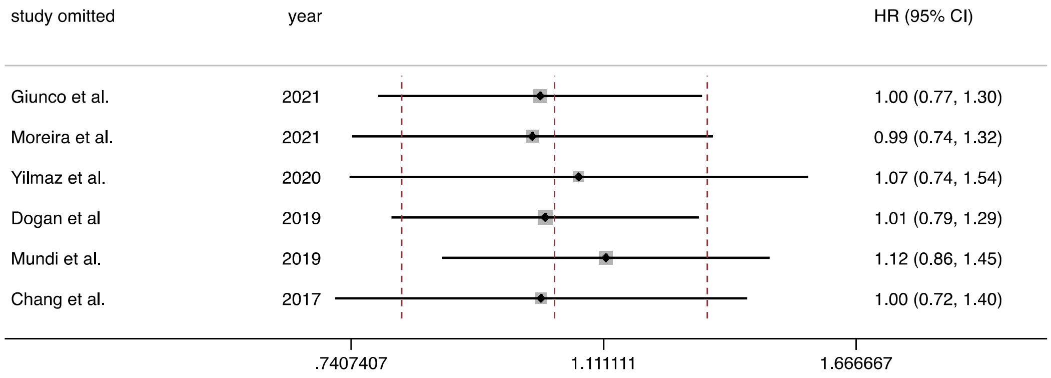

Figure 18. Interval plot graphically representing the sensitivity analysis of the studies pooled in the meta-analysis on the association between TERT mutations and overall survival in OSCC. TERT, telomerase reverse transcriptase; OSCC, oral squamous cell carcinoma; HR, hazard ratio; CI, confidence intervals.

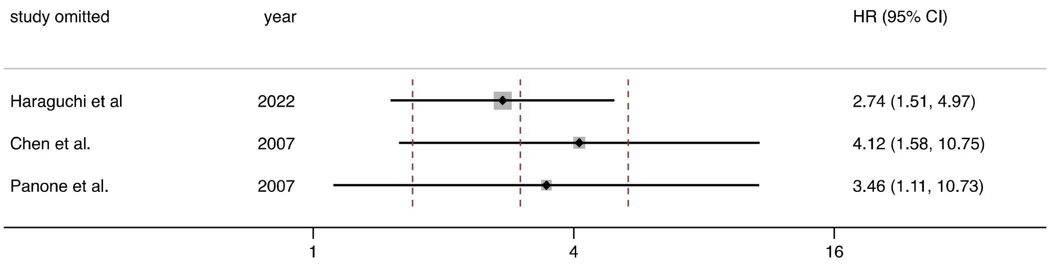

Figure S19. Interval plot graphically representing the sensitivity analysis of the studies pooled in the meta-analysis on the association between TERT protein overexpression and overall survival in OSCC.Sensitivity analysis ("leave-one-out" method) of the meta-analysis results, sequentially omitting one study at a time. TERT, telomerase reverse transcriptase; OSCC, oral squamous cell carcinoma; HR, hazard ratio; CI, confidence intervals.

10.2. TERT upregulation and disease-free survival in OSCC

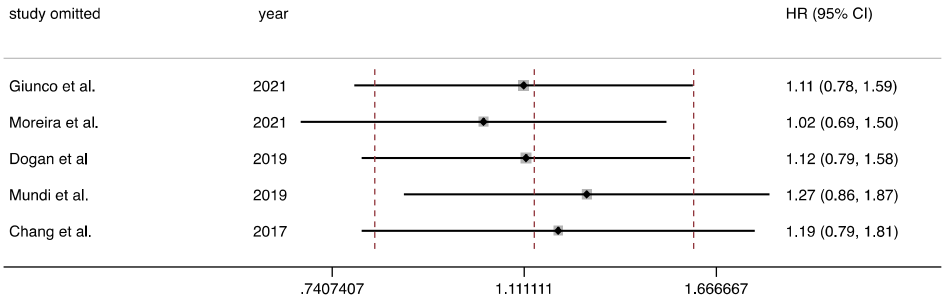

Figure S20. Interval plot graphically representing the sensitivity analysis of the studies pooled in the meta-analysis on the association between TERT mutations and disease-free survival in OSCC. TERT, telomerase reverse transcriptase; OSCC, oral squamous cell carcinoma; HR, hazard ratio; CI, confidence intervals.

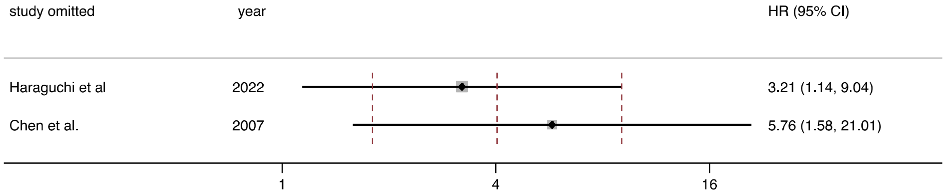

Figure S21. Interval plot graphically representing the sensitivity analysis of the studies pooled in the meta-analysis on the association between TERT protein overexpression and disease-free survival in OSCC. Sensitivity analysis ("leave-one-out" method) of the meta-analysis results, sequentially omitting one study at a time. TERT, telomerase reverse transcriptase; OSCC, oral squamous cell carcinoma; HR, hazard ratio; CI, confidence intervals.

10.3. TERT upregulation and T status in OSCC

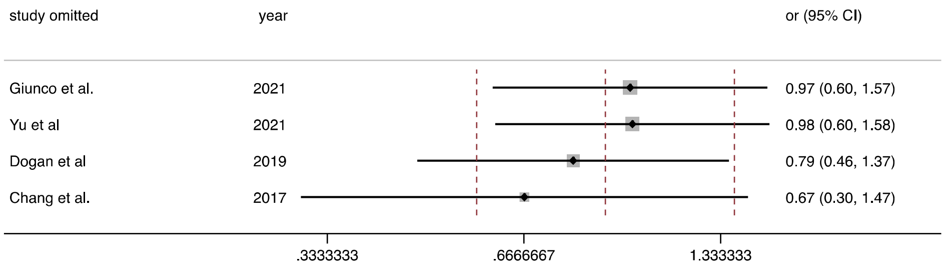

Figure S22. Interval plot graphically representing the sensitivity analysis of the studies pooled in the meta-analysis on the association between TERT mutations and T status in OSCC. TERT, telomerase reverse transcriptase; OSCC, oral squamous cell carcinoma; or, odds ratio; CI, confidence intervals.

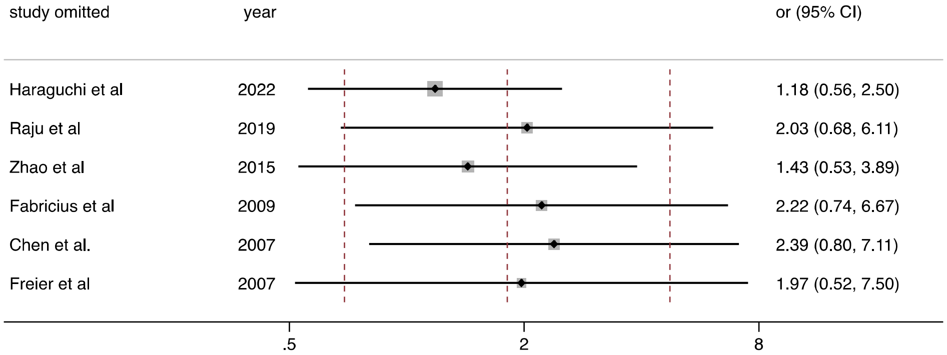

Figure S23. Interval plot graphically representing the sensitivity analysis of the studies pooled in the meta-analysis on the association between TERT protein overexpression and T status in OSCC. Sensitivity analysis ("leave-one-out" method) of the meta-analysis results, sequentially omitting one study at a time. TERT, telomerase reverse transcriptase; OSCC, oral squamous cell carcinoma; or, odds ratio; CI, confidence intervals.

10.4. TERT upregulation and N status in OSCC

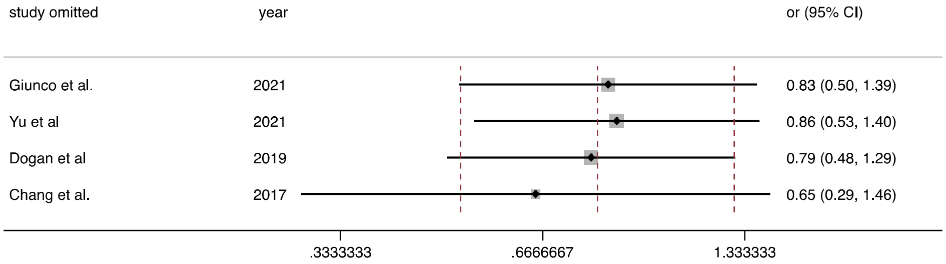

Figure S24. Interval plot graphically representing the sensitivity analysis of the studies pooled in the meta-analysis on the association between TERT mutations and N status in OSCC. TERT, telomerase reverse transcriptase; OSCC, oral squamous cell carcinoma; or, odds ratio; CI, confidence intervals.

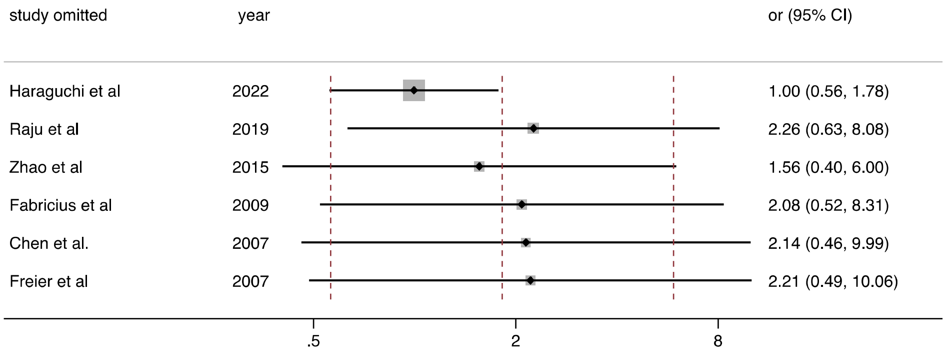

Figure S25. Interval plot graphically representing the sensitivity analysis of the studies pooled in the meta-analysis on the association between TERT protein overexpression and N status in OSCC. Sensitivity analysis ("leave-one-out" method) of the meta-analysis results, sequentially omitting one study at a time. TERT, telomerase reverse transcriptase; OSCC, oral squamous cell carcinoma; or, odds ratio; CI, confidence intervals.

10.5. TERT upregulation and clinical stage in OSCC

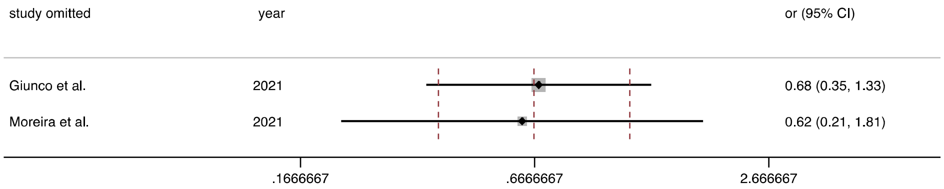

Figure S26. Interval plot graphically representing the sensitivity analysis of the studies pooled in the meta-analysis on the association between TERT mutations and clinical stage in OSCC. TERT, telomerase reverse transcriptase; OSCC, oral squamous cell carcinoma; or, odds ratio; CI, confidence intervals.

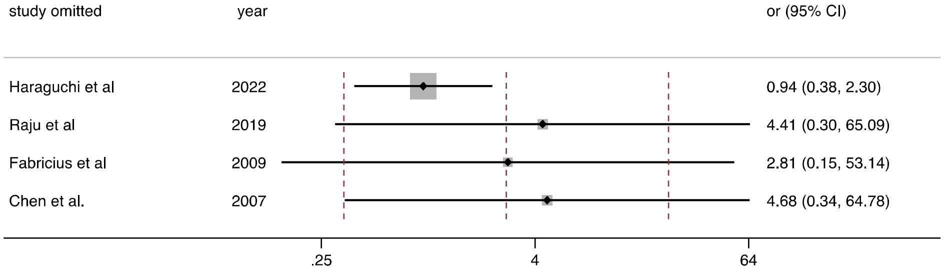

Figure S27. Interval plot graphically representing the sensitivity analysis of the studies pooled in the meta-analysis on the association between TERT protein overexpression and clinical stage in OSCC. Sensitivity analysis ("leave-one-out" method) of the meta-analysis results, sequentially omitting one study at a time. TERT, telomerase reverse transcriptase; OSCC, oral squamous cell carcinoma; or, odds ratio; CI, confidence intervals.

10.6. TERT upregulation and histological grade in OSCC

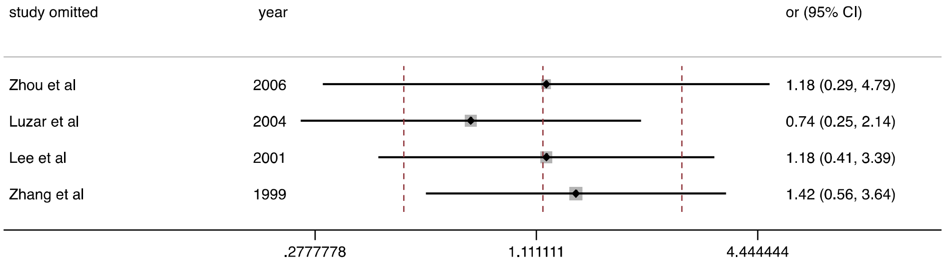

Figure S28. Interval plot graphically representing the sensitivity analysis of the studies pooled in the meta-analysis on the association between TERT mRNA overexpression and histological grade in OSCC. TERT, telomerase reverse transcriptase; OSCC, oral squamous cell carcinoma; or, odds ratio; CI, confidence intervals.

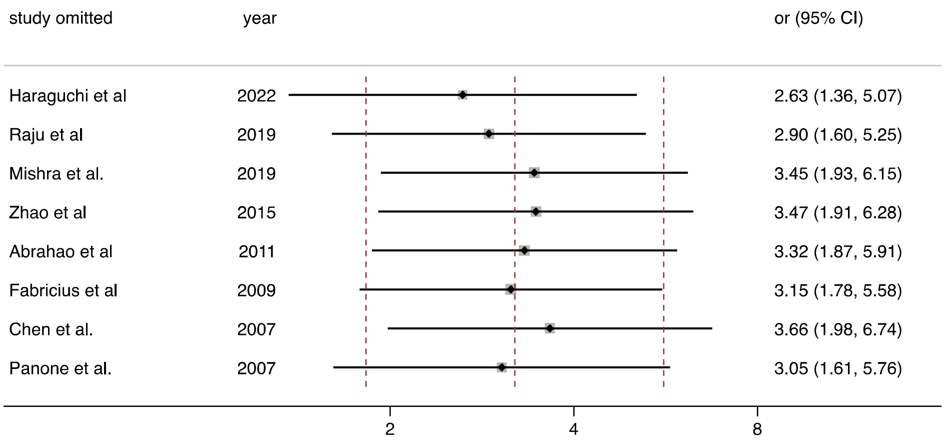

Figure S29. Interval plot graphically representing the sensitivity analysis of the studies pooled in the meta-analysis on the association between TERT protein overexpression and histological grade in OSCC. Sensitivity analysis ("leave-one-out" method) of the meta-analysis results, sequentially omitting one study at a time. TERT, telomerase reverse transcriptase; OSCC, oral squamous cell carcinoma; or, odds ratio; CI, confidence intervals.

11. Analysis of secondary clinico-pathological variables

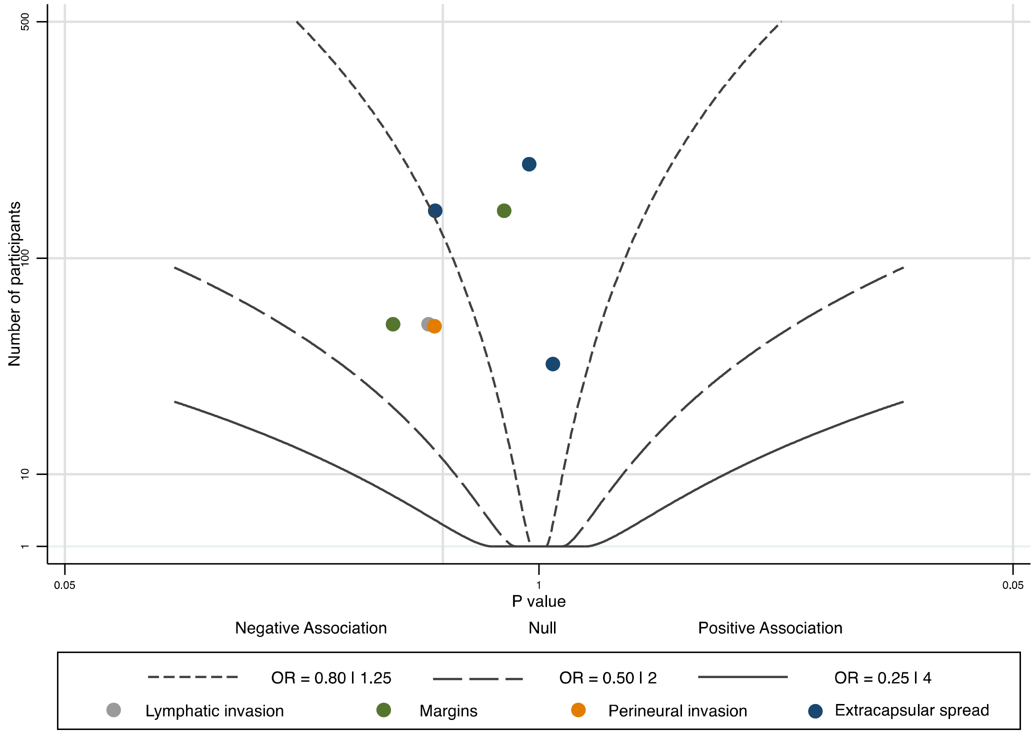

Figure S30. Albatross plot graphically representing the association between TERT upregulation (all were gene mutations) and secondary clinicopathological parameters rarely reported in primary-level studies, but harbouring relevant prognostic implications. Primary-level studies were represented by a circle of different colours, according to the clinicopathological parameter investigated (see legend). Two-sides p-values (horizontal x-axis) with results separated according to positive/negative association (i.e., the observed direction of effect) were plotted against the number of participants included within each study (vertical y-axis). The albatross plot allows a better interpretation of p-values from the variables that did not enter in the meta-analysis, in the context of the study sample sizes. Small studies lie toward the bottom of the plot and large studies toward the top. Effect contours (black continuous and intermittent lines) were drawn on the plot showing the ranges of the magnitudes of effect, using odds ratios (OR). A p-value < 0.05 was considered significant.

12. List of excluded studies with reasons

No clinico-pathological outcomes (n=12)

Annunziata, C., Pezzuto, F., Greggi, S., Ionna, F., Losito, S., Botti, G., Buonaguro, L., Buonaguro, F. M., & Tornesello, M. L. (2018). Distinct profiles of TERT promoter mutations and telomerase expression in head and neck cancer and cervical carcinoma. *International journal of cancer*, *143*(5), 1153–1161. https://doi.org/10.1002/ijc.31412

Miyazaki, Y., Yoshida, N., Nozaki, T., Inoue, H., Kikuchi, K., & Kusama, K. (2015). Telomerase activity in the occurrence and progression of oral squamous cell carcinoma. *Journal of oral science*, *57*(4), 295–303. https://doi.org/10.2334/josnusd.57.295

Li, Y. F., Hsiao, Y. H., Lai, Y. H., Chen, Y. C., Chen, Y. J., Chou, J. L., Chan, M. W., Lin, Y. H., Tsou, Y. A., Tsai, M. H., & Tai, C. K. (2015). DNA methylation profiles and biomarkers of oral squamous cell carcinoma. *Epigenetics*, *10*(3), 229–236. https://doi.org/10.1080/15592294.2015.1006506

Alawi, F., Lin, P., Ziober, B., & Patel, R. (2011). Correlation of dyskerin expression with active proliferation independent of telomerase. *Head & neck*, *33*(7), 1041–1051. https://doi.org/10.1002/hed.21579

Kumar, S. K., Zain, R. B., Ismail, S. M., & Cheong, S. C. (2005). Human telomerase reverse transcriptase expression in oral carcinogenesis--a preliminary report. *Journal of experimental & clinical cancer research : CR*, *24*(4), 639–646.

Wu, G. Y., Zhu, L., Feng, Z. Q., Peng, T., & Qi, B. (2005). *Shanghai kou qiang yi xue = Shanghai journal of stomatology*, *14*(1), 51–54.

Liao, C. T., Chen, I. H., Chang, J. T., Wang, H. M., Hsieh, L. L., & Cheng, A. J. (2003). Lack of correlation of betel nut chewing, tobacco smoking, and alcohol consumption with telomerase activity and the severity of oral cancer. *Chang Gung medical journal*, *26*(9), 637–645.

Downey, M. G., Going, J. J., Stuart, R. C., & Keith, W. N. (2001). Expression of telomerase RNA in oesophageal and oral cancer. *Journal of oral pathology & medicine : official publication of the International Association of Oral Pathologists and the American Academy of Oral Pathology*, *30*(10), 577–581. https://doi.org/10.1034/j.1600-0714.2001.301001.x

Kim, H. R., Christensen, R., Park, N. H., Sapp, P., Kang, M. K., & Park, N. H. (2001). Elevated expression of hTERT is associated with dysplastic cell transformation during human oral carcinogenesis in situ. *Clinical cancer research : an official journal of the American Association for Cancer Research*, *7*(10), 3079–3086.

Fujimoto, R., Kamata, N., Yokoyama, K., Ueda, N., Satomura, K., Hayashi, E., & Nagayama, M. (2001). Expression of telomerase components in oral keratinocytes and squamous cell carcinomas. *Oral oncology*, *37*(2), 132–140. https://doi.org/10.1016/s1368-8375(00)00073-7

Koscielny, S., Fiedler, W., Dahse, R., & Beleites, E. (2000). Reaktivierung der Telomerase in Plattenepithelkarzinomen des Kopf-Hals-Bereiches [Reactivation of telomerase in squamous epithelial carcinomas in the area of the head and neck]. *Laryngo- rhino- otologie*, *79*(9), 551–556. https://doi.org/10.1055/s-2000-6937

Zhang, L., Zhang, W., & Liu, Z. (1999). *Hua xi kou qiang yi xue za zhi = Huaxi kouqiang yixue zazhi = West China journal of stomatology*, *17*(4), 307–309.

Lack of essential data (n=6)

Yoon, A. J., Santella, R. M., Wang, S., Kutler, D. I., Carvajal, R. D., Philipone, E., Wang, T., Peters, S. M., Stewart, C. R., Momen-Heravi, F., Troob, S., Levin, M., AkhavanAghdam, Z., Shackelford, A. J., Canterbury, C. R., Shimonosono, M., Hernandez, B. Y., McDowell, B. D., & Nakagawa, H. (2021). MicroRNA-Based Cancer Mortality Risk Scoring System and hTERT Expression in Early-Stage Oral Squamous Cell Carcinoma. *Journal of oncology*, *2021*, 8292453. https://doi.org/10.1155/2021/8292453

Gissi, D. B., Fabbri, V. P., Gabusi, A., Lenzi, J., Morandi, L., Melotti, S., Asioli, S., Tarsitano, A., Balbi, T., Marchetti, C., & Montebugnoli, L. (2020). Pre-Operative Evaluation of DNA Methylation Profile in Oral Squamous Cell Carcinoma Can Predict Tumor Aggressive Potential. *International journal of molecular sciences*, *21*(18), 6691. https://doi.org/10.3390/ijms21186691

Vinothkumar, V., Arunkumar, G., Revathidevi, S., Arun, K., Manikandan, M., Rao, A. K., Rajkumar, K. S., Ajay, C., Rajaraman, R., Ramani, R., Murugan, A. K., & Munirajan, A. K. (2016). TERT promoter hot spot mutations are frequent in Indian cervical and oral squamous cell carcinomas. *Tumour biology : the journal of the International Society for Oncodevelopmental Biology and Medicine*, *37*(6), 7907–7913. https://doi.org/10.1007/s13277-015-4694-2

Carkic, J., Nikolic, N., Radojevic-Skodric, S., Kuzmanovic-Pficer, J., Brajovic, G., Antunovic, M., Milasin, J., & Popovic, B. (2016). The role of TERT-CLPTM1L SNPs, hTERT expression and telomere length in the pathogenesis of oral squamous cell carcinoma. *Journal of oral science*, *58*(4), 449–458. https://doi.org/10.2334/josnusd.16-0108

Palani, J., Lakshminarayanan, V., & Kannan, R. (2011). Immunohistochemical detection of human telomerase reverse transcriptase in oral cancer and pre-cancer. *Indian journal of dental research : official publication of Indian Society for Dental Research*, *22*(2), 362. https://doi.org/10.4103/0970-9290.84281

Fujita, H., Nagata, M., Hoshina, H., Nagashima, K., Seki, Y., Tanaka, K., Nishizawa, R., Shingaki, S., Ohnishi, M., & Takagi, R. (2004). Clinical significance and usefulness of quantification of telomerase activity in oral malignant and nonmalignant lesions. *International journal of oral and maxillofacial surgery*, *33*(7), 693–699. https://doi.org/10.1016/j.ijom.2004.01.016

Overlapping populations (n=2)

Boscolo-Rizzo, P., Giunco, S., Rampazzo, E., Brutti, M., Spinato, G., Menegaldo, A., Stellin, M., Mantovani, M., Bandolin, L., Rossi, M., Del Mistro, A., Tirelli, G., Dei Tos, A. P., Guerriero, A., Niero, M., Da Mosto, M. C., Polesel, J., & De Rossi, A. (2020). TERT promoter hotspot mutations and their relationship with TERT levels and telomere erosion in patients with head and neck squamous cell carcinoma. *Journal of cancer research and clinical oncology*, *146*(2), 381–389. https://doi.org/10.1007/s00432-020-03130-z

Boscolo-Rizzo, P., Rampazzo, E., Perissinotto, E., Piano, M. A., Giunco, S., Baboci, L., Spinato, G., Spinato, R., Tirelli, G., Da Mosto, M. C., Del Mistro, A., & De Rossi, A. (2015). Telomere shortening in mucosa surrounding the tumor: biosensor of field cancerization and prognostic marker of mucosal failure in head and neck squamous cell carcinoma. *Oral oncology*, *51*(5), 500–507. https://doi.org/10.1016/j.oraloncology.2015.02.100

13. Validation of methodological quality

Table S3. AMSTAR2 scoring system

Tool	Study design	Items 1	2	3	4	5	6	7	8	9	10	11	12	13	14	15	16	Overall rating	Score
**AMSTAR2**	**Systematic review and meta-analysis**																	HIGH	16

Explanation: The methodological quality of this systematic review followed the *Assesing the Methodological Quality of Systematic Reviews-2* (AMSTAR2) recommendations and was validated using this tool. AMSTAR2 was designed to develop, evaluate and validate high quality systematic reviews through 16 items. An overall rating is obtained based on weaknesses(*) in the following critical and non-critical items (the checklist was also included in the precedent appendix page):

1. Did the research questions and inclusion criteria for the review include the components of PICO?
2. Did the report of the review contain an explicit statement that the review methods were established prior to the conduct of their review, and did the report justify any significant deviations from the protocol?*
3. Did the review authors explain their selection of the study designs for inclusion in the review?
4. Did the review authors use a comprehensive literature search strategy?*
5. Did the review authors perform study selection in duplicate?
6. Did the review authors perform data extraction in duplicate?
7. Did the review authors provide a list of excluded studies and justify the exclusions?*
8. Did the review authors describe the included studies in adequate detail?
9. Did the review authors use a satisfactory technique for assessing the risk of bias (RoB) in individual studies that were included in the review?*
10. Did the review authors report on the sources of funding for the studies included in the review?
11. If meta-analysis was performed, did the review authors use appropriate methods for statistical combination of results?*
12. If meta-analysis was performed, did the review authors assess the potential impact of RoB in individual studies on the results of the metaanalysis or other evidence synthesis?
13. Did the review authors account for RoB in individual studies when interpreting/discussing the results of the review?*
14. Did the review authors provide a satisfactory explanation for, and discussion of, any heterogeneity observed in the results of the review?
15. If they performed quantitative synthesis, did the review authors carry out an adequate investigation of publication bias (small study bias) and discuss its likely impact on the results of the review?*
16. Did the review authors report any potential sources of conflict of interest, including any funding they received for conducting the review?

High overall rating: No or one non-critical weakness. The systematic review provides an accurate and comprehensive summary of the results of the available studies that address the question of interest.
